# Metabolic reprogramming in ischemic stroke: when glycolytic overdrive meets lipid storm

**DOI:** 10.1038/s41419-025-08114-w

**Published:** 2025-11-03

**Authors:** Yuchun Wang, Minyan Ge, Jinling Wang, Yiming Xu, Nianhong Wang, Shumao Xu

**Affiliations:** 1 Department of Rehabilitation Medicine, Huashan Hospital, Institute of Science and Technology for Brain-inspired Intelligence (ISTBI), Fudan University, Shanghai, China; 2 National Center for Neurological Disorders, Shanghai, China; 3 National Clinical Research Center for Geriatric Diseases, Shanghai, China; 4 Max-Planck Institute for Solid State Research, Stuttgart, Germany

**Keywords:** Cell death and immune response, Calcium and phosphate metabolic disorders, Predictive markers

## Abstract

Ischemic stroke, a leading cause of global disability and mortality, remains inadequately treated beyond reperfusion, with persistent translational failures in neuroprotection. We posit metabolic reprogramming in ischemic stroke (MRIS) as the unifying pathophysiological driver, where acute compensatory glycolysis collides with enzymatic lipid peroxidation to ignite neuroinflammation and early deficits. This metabolic crisis transcends neuron-centric models, integrating single-cell heterogeneity with bidirectional brain-peripheral crosstalk: hepatic ketogenesis releases neuroprotective β-hydroxybutyrate; adipose lipolysis fuels inflammatory storms; and gut dysbiosis disrupts barrier integrity, amplifying neuroinflammation. MRIS progresses through temporally stratified phases. An acute glycolytic-excitotoxic crisis and nicotinamide adenine dinucleotide (NAD^+^) depletion trigger neuroimmune dysfunction. Subacute lipid peroxidation cascades trigger ferroptosis and microglial polarization, whereas chronic-phase recovery of executive networks is scaffolded by sirtuin-mediated mitochondrial biogenesis and the interplay between adenosine monophosphate-activated protein kinase (AMPK) and mechanistic target of rapamycin (mTOR). Spatial metabolomics and single-cell omics decode cell-type-specific vulnerabilities, revealing astrocytic lipid droplets, microglial succinate accumulation, and neuron-glia lactate shuttles as targetable nodes. Chronobiology further dictates therapeutic windows: lactate dehydrogenase A (LDHA) inhibition mitigates hyperacute acidosis, while NAD^+^ salvage pathways optimize chronic mitochondrial plasticity. We propose that metabolic reprogramming is a central amplifier of both ischemic injury and recovery, linking cerebral vascular occlusion to systemic organ dysfunction. By reframing stroke within a vascular-metabolic continuum, MRIS shifts the paradigm from a neuron-centric view to one of systemic bioenergetic failure, accounting for past translational gaps and opening pathways for precision therapies, from pentose phosphate pathway modulation to nanoparticle-based metabolite delivery and microbiome interventions. In this framework, metabolic plasticity becomes not just a consequence but a therapeutic target, transforming stroke from an untreatable vascular event to a modifiable metabolic disorder.

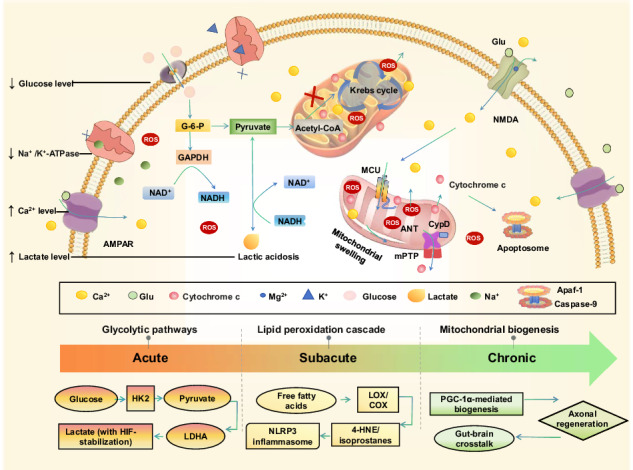

## Introduction

Ischemic stroke transcends its identity as a vascular emergency, emerging instead as a systemic metabolic epicenter where cellular survival strategies collide with catastrophic systemic dysregulation, irrevocably reshaping human behavior, cognition, and psychosocial resilience [1–5]. Central to this transformation is metabolic reprogramming in ischemic stroke (MRIS), the fundamental driver and organizing paradigm of the injury-recovery continuum. MRIS constitutes a dynamic recalibration of energy metabolism across neurons, glia, vascular cells, and brain-peripheral organ axes, entailing systematic rewiring of cellular bioenergetics including shifts in glucose/fatty acid oxidation (FAO) stoichiometry, altered nicotinamide adenine dinucleotide (NAD^+^)/ nicotinamide adenine dinucleotide (NADH) redox balance, and mitochondrial quality control adaptations to meet ischemic energetic demands while navigating metabolic constraints [6–15]. This metabolic metamorphosis, spanning milliseconds to months, recasts stroke as a disorder of maladaptive resilience, where disrupted energy trade-offs shape neurobehavioral outcomes ranging from motor deficits to cognitive dysfunction [1].

The ischemic cascade initiates a metabolic triage with profound behavioral consequences. Within seconds, neuronal adenosine triphosphate (ATP) depletion triggers a glycolytic surge flooding cells with lactate, destabilizing pH, and paralyzing ion pumps, a biochemical crisis manifesting as cytotoxic edema and acute cognitive-motor dysfunction [16]. Crucially, this crisis extends beyond energy failure: phospholipase-driven lipid membrane degradation unleashes free fatty acids that oxidize mitochondria, permeabilize lysosomes, and ignite pyrin domain-containing protein 3 (NLRP3) inflammasomes [17–19]. This glycolytic exhaustion, intertwined with lipid toxicity, coalesces into a self-reinforcing metabolic vortex that etches both the penumbra’s fate and the patient’s long-term functional identity, directly linking metabolic collapse to neuropsychiatric sequelae like post-stroke depression and anxiety [19, 20].

Astrocytes, traditionally passive supporters, emerge as metabolic arbitrators. During glucose deprivation, astrocytes repurpose lipolytic fragments into lipid droplets via diacylglycerol acyltransferase (DGAT)-catalyzed esterification and perilipin-2 (PLIN2)-mediated stabilization, sequestering cytotoxic fatty acids to protect neurons from lipotoxicity [21–36] (Table 1). Microglia undergo metabolic reinvention: early succinate accumulation drives inflammatory rage, yet later oxidation of this metabolite switches them to anti-inflammatory phagocytes [37]. Beyond the brain, systemic actors engage in this metabolic ballet through bidirectional axes. Gut-derived short-chain fatty acids (SCFAs) like butyrate strengthen blood-brain barrier (BBB) integrity via histone deacetylase (HDAC) inhibition, suppressing nuclear factor kappa B (NF-κB)-driven inflammation [9–12, 38]. Post-stroke, gut dysbiosis reduces butyrate production, increasing BBB permeability and microglial activation [39]. Conversely, β-hydroxybutyrate from hepatic ketogenesis bypasses glycolytic impairment, directly fueling neuronal mitochondria via monocarboxylate transporter (MCT) [40]. While these gut-liver-brain feedback loops are mechanistically plausible, preclinical evidence suggests their potential involvement in ischemia; we highlight them as testable hypotheses central to the MRIS framework [13].

**Table 1:** Metabolic reprogramming in ischemic stroke across neural and systemic compartments.

Cell type/organ	Metabolic reprogramming	Mediators/pathways	Impact on the MRIS framework	Downstream effects
**Neurons**	Glycolytic collapse → NAD^+^ salvage failure [27]	PARP-1 [29], NAMPT [24], sirtuin-1 [28]	Initiates an excitotoxic cascade and energy crisis	ATP depletion, ferroptosis, synaptic failure
**Astrocytes**	Glycogenolysis↑ → lipid droplet accumulation	HIF-1α/PDK, DGAT1, PLIN2 [34, 35]	Fuel neuroinflammation via PGE2 release	Microglial M1 polarization, lactic acidosis
**Microglia**	Succinate accumulation → FAO dominance	SDH, CPT1A, NLRP3 [32, 33]	Drive NLRP3 inflammasome via ROS overproduction	BBB leakage, neutrophil recruitment
**Endothelial cells**	Glycolysis → fatty acid oxidation	HIF-2α, CPT1A, ROCK [30]	Impair BBB integrity and vasodilation	Thrombus formation, immune cell infiltration
**Pericytes**	Lactate-fueled contraction	ASIC activation → RhoA/ROCK signaling [31]	Cause “no-reflow” post-recanalization	Penumbral hypoperfusion, metabolic crisis
**Liver**	Ketogenesis ↑ → BHB neuroprotection	CPT1A, HMGCS2 [25]	Provide a neuroprotective alternative fuel	Reduced ROS, preserved mitochondrial function
**Adipose tissue**	Lipolysis ↑ → FFA release	ATGL, HSL [36]	Amplify systemic lipid toxicity	NLRP3 activation, insulin resistance
**Gut**	Dysbiosis → SCFA ↓	Akkermansia depletion → butyrate ↓ [26]	Disrupt gut-brain signaling	Microglial priming, BBB leakage

Early therapeutic interventions target the temporal duality of MRIS, aiming to mitigate the initial uncompensated glycolytic acceleration (or glycolytic frenzy) by inhibiting lactate dehydrogenase to prevent acidosis [41]. Subsequent strategies focus on enhancing ketolysis or delivering tricarboxylic acid (TCA) cycle intermediates to reactivate stalled mitochondria. Emerging chronotherapeutic approaches acknowledge circadian governance of mitochondrial biogenesis and antioxidant defenses [42]. Meanwhile, single-cell metabolomics reveals startling heterogeneity: adjacent neurons embark on divergent metabolic trajectories, with some succumbing to ferroptosis while others activate salvage pathways via purine recycling [43, 44]. Sirtuin-driven mitochondrial biogenesis, together with a balancing act between adenosine monophosphate-activated protein kinase (AMPK) and mechanistic target of rapamycin (mTOR) signaling, scaffolds functional recovery, positioning NAD^+^ salvage as a therapeutic linchpin [45]. This metabolic lens reveals unexpected bridges to systemic disease. The same glycolytic-lipid axis driving stroke pathology resurfaces in diabetic vasculopathy and atherosclerotic plaque rupture, suggesting conserved therapeutic targets [46]. The gut microbiome further modulates MRIS progression via metabolites like trimethylamine *N*-oxide (TMAO), influencing platelet reactivity [13]. Therefore, while ischemic stroke originates from vascular occlusion (thromboembolic, atherosclerotic, or hemodynamic), triggering metabolic adaptations, and vascular recanalization remains foundational, metabolic reprogramming extends the injury/recovery continuum decisively beyond mere perfusion restoration. Collectively, we propose MRIS as a central amplifier of ischemic injury and recovery that bridges vascular occlusion to systemic organ dysfunction, positioning it as a vascular-metabolic continuum.

As the field advances, pressing questions emerge: Can we develop metabolic biomarkers that distinguish salvageable penumbra from core infarct? Can dietary or circadian interventions enhance metabolic flexibility to precondition recovery in high-risk populations? And crucially, how do aging and comorbidities hijack MRIS pathways, explaining stroke’s clinical heterogeneity [47, 48]? By interrogating the intersections of metabolism, immunity, and neural circuitry, we advance toward therapies that not only salvage neurons but also reprogram their survival pathways, revealing how maladaptive metabolic responses in stroke can be therapeutically redirected toward resilience. In this review, we unravel MRIS as a unifying framework for ischemic stroke pathophysiology. We explore how the convergence of compensatory glycolysis (acute glycolytic acceleration) and lipid peroxidation cascades (characterized by pro-inflammatory mediator release) dictates neuronal fate through immunometabolic checkpoints. Glycolytic acceleration generates succinate accumulation, which inhibits succinate dehydrogenase (SDH), stabilizing hypoxia-inducible factor 1 alpha (HIF-1α) to drive pro-inflammatory microglial polarization. Conversely, lipid peroxidation cascades, non-apoptotic cell death driven by iron-dependent phospholipid peroxidation involving arachidonate LOX-15 (15-lipoxygenase), glutathione peroxidase 4 (GPX4), polyunsaturated fatty acid-containing phospholipids, and Fe^2+^ trigger itaconate production. Itaconate activates the nuclear factor erythroid factor 2 (NRF2) pathway via Kelch-like erythroid cell-derived homology-associated protein 1 (KEAP1) alkylation, suppressing NLRP3 inflammasomes and promoting anti-inflammatory responses. By synthesizing spatial metabolomics (mapping regional lipid peroxide gradients), single-cell transcriptomics (resolving astrocyte-microglia crosstalk), and chronobiology (timing interventions to metabolic rhythms), we uncover therapeutic strategies to reprogram MRIS from maladaptation toward resilience.

## MRIS: from neurons to systemic biology

MRIS extends beyond neurons, astrocytes, and microvasculature, emerging as a systemic metabolic network that redefines stroke from a localized neurovascular event to a whole-body bioenergetic disorder. This positions MRIS as a unifying framework linking cerebral injury to peripheral organ dysfunction through coordinated metabolic crosstalk. Single-nucleus RNA sequencing identifies nine major cell types, including vascular leptomeningeal cells and oligodendrocyte precursor cells [49]. Excitatory neurons, dominant in gray matter voxels with high oxidative phosphorylation demand, exhibit tight complex I/complex IV coupling for ATP synthesis yet remain vulnerable to glutamate excitotoxicity. Microglia display discordant complex I/complex IV ratios signaling NF-κB-driven M1 polarization, while astrocytes shift toward glycolysis, exacerbating lactate accumulation and BBB compromise [50].

The liver-brain axis delivers critical adaptations: hepatic β-hydroxybutyrate production provides neuroprotective fuel that bypasses glycolytic collapse [10, 51], while impaired urea cycle function induces hyperammonemia, exacerbating astrocytic glutamine synthetase burden and cerebral edema [52]. Simultaneously, the adipose tissue-inflammasome axis amplifies injury: lipolysis releases free fatty acids that potentiate microglial NLRP3 inflammasome activation via Toll-like receptor 4 (TLR4) signaling [53, 54] and adipokine imbalance (leptin resistance/adiponectin deficiency) disrupts hypothalamic satiety circuits, mechanistically linking post-stroke depression to adipose dysfunction [55]. The gut-brain axis contributes through dysbiosis: stroke-induced sympathetic surges compromise gut barrier integrity, permitting endotoxin translocation that primes microglia via TLR4 [56]. Concomitant short-chain fatty acid deficits, particularly butyrate loss from Akkermansia muciniphila depletion, impair BBB claudin-5 expression and neuronal mitochondrial biogenesis [57, 58]. Conversely, the skeletal muscle-myokine axis supports recovery: exercise-responsive irisin enhances brain-derived neurotrophic factor expression to promote axonal regeneration, with post-stroke depletion worsening motor outcomes [59].

Aging imposes metabolic inflexibility on MRIS responses: mitochondrial biogenesis declines due to peroxisome proliferator-activated receptor gamma coactivator 1 alpha suppression, while nicotinamide adenine dinucleotide depletion impairs sirtuin-mediated stress adaptation [60–62] (Table S1). In aged astrocytes, lipolytic defects (e.g., reduced adipose-triglyceride lipase activity) exacerbate lipid droplet accumulation, converting protective storage into neurotoxic reservoirs. Microglia develop phenotypes with heightened succinate-driven NLRP3 activation and impaired phagocytosis, amplifying post-stroke neuroinflammation [63].

Metabolic master switches orchestrate MRIS pathophysiology. AMPK, mTOR, and sirtuins serve as evolutionary gatekeepers of cellular metabolism, orchestrating distinct phases of MRIS pathophysiology. During acute ischemia, energy depletion activates AMPK, upregulating key glycolytic enzymes including hexokinase 2 (HK2), lactate dehydrogenase A (LDHA), and MCT4 to drive compensatory glycolysis for rapid ATP generation, while simultaneously suppressing mTOR-mediated anabolic processes to conserve cellular resources [11, 64, 65] (Table 2). AMP-activated protein kinase, mechanistic target of rapamycin, and sirtuins serve as evolutionary gatekeepers: acute ischemia triggers AMP-activated protein kinase activation to accelerate emergency glycolysis while inhibiting a mechanistic target of rapamycin to conserve energy [66]. In the chronic phase, mTOR dominance emerges, promoting lipid synthesis that fuels neuroinflammatory cascades [67]. Concurrently, NAD^+^ depletion, exacerbated by poly(ADP-ribose) polymerase-1 (PARP-1) hyperactivation, cripples sirtuin activity, impairing mitochondrial biogenesis via peroxisome proliferator-activated receptor gamma coactivator 1 alpha (PGC-1α) dysregulation and reducing antioxidant defense through Forkhead box O (FOXO) signaling. This triad-mediated transition from acute glycolytic adaptation to chronic lipid dysregulation presents time-sensitive therapeutic targets for interrupting the MRIS cascade [68, 69]. Chronic phases exhibit mechanistic target of rapamycin dominance, promoting lipid synthesis that fuels neuroinflammation. Nicotinamide adenine dinucleotide depletion exacerbated by PARP-1 hyperactivation cripples sirtuin activity [70], impairing mitochondrial repair. This triad governs the transition from glycolytic crisis to lipid storm, presenting druggable nodes for time-sensitive interventions.

**Table 2:** Phase-specific MRIS pathophysiology, biomarkers, and precision therapeutics.

Phase	Metabolic events	Systemic consequences	Key biomarkers	Validated therapeutics	Comorbidity adjustments	Validating evidence
**Acute**	Glycolytic surge → lactate acidosis; Glutamate excitotoxicity → Ca^2+^ overload; PARP-1 hyperactivation → NAD^+^ depletion	Hepatic ketogenesis ↑ (BHB neuroprotection)	CSF lactate >4 mM; Serum glutamate >100 μM	NMDA antagonists (nerinetide); PARP-1 inhibitors; LDHA inhibitors	Withhold metformin (diabetics); Renal dose adjustment	BHB infusion ↓ infarct 40% [11]
**Subacute**	Lipid peroxidation → ferroptosis; HIF-1α-driven neuroinflammation; Microglial M1 → M2 transition	Gut dysbiosis (Akkermansia ↓), SCFAs ↓	Plasma 4-HNE > 2 μM; ACSL4 upregulation; Mitochondrial shrinkage; lipid peroxidation magnetic resonance imaging (single-cell analyses)	Ferroptosis inhibitors (liproxstatin-1); HIF-1α modulators (roxadustat); NLRP3 inhibitors (MCC950)	Avoid in liver disease (CYP3A4 risk); Thrombocytopenia dose reduction	Fecal transplant ↑ SCFAs → outcome improvement [64]
**Chronic**	Mitochondrial biogenesis ↑; Axonal regeneration; Gut-brain axis rebalancing	Muscle atrophy (irisin ↓), cardiac dysfunction	Serum BHB > 0.5 mM; Fecal Akkermansia abundance	NAD^+^ boosters (nicotinamide riboside); DGAT1 inhibitors; Akkermansia probiotics	Probiotic contraindicated in IBD; ↓ NAD^+^ booster dose in the elderly	Serum irisin predicts motor recovery [65]

Mechanistically, the glycolytic-lipid axis drives injury progression. Acute glycolytic flux generates lactate that acidifies the microenvironment, proton loads inactivating sirtuin deacetylases to impair peroxisome proliferator-activated receptor gamma coactivator 1 alpha-mediated mitochondrial biogenesis [71] while activating calcium-independent phospholipase A_2_ to liberate arachidonic acid [72]. Arachidonic acid is metabolized by cyclooxygenase-2 into pro-inflammatory prostaglandins and by LOX-15 into lipid peroxides that initiate ferroptosis [73]. Single-cell lipidomics confirms co-localization of MCT4 and LOX-15 in peri-infarct neurons [49]. Systemically, lipid peroxides (4-hydroxynonenal, isoprostanes) disrupt the BBB via matrix metalloproteinase-9 (MMP-9) activation [74], permitting peripheral free fatty acid influx (body mass index >30) associated with 3-fold higher cerebrospinal fluid 4-hydroxynonenal [75] and gut-endotoxin translocation. Lipopolysaccharide binding activates microglial TLR4-NLRP3 inflammasomes, releasing interleukin (IL) 1β [76], a feedforward loop where systemic inflammation further suppresses astrocytic β-oxidation, worsening lipid toxicity [77].

**Fig. 1:**
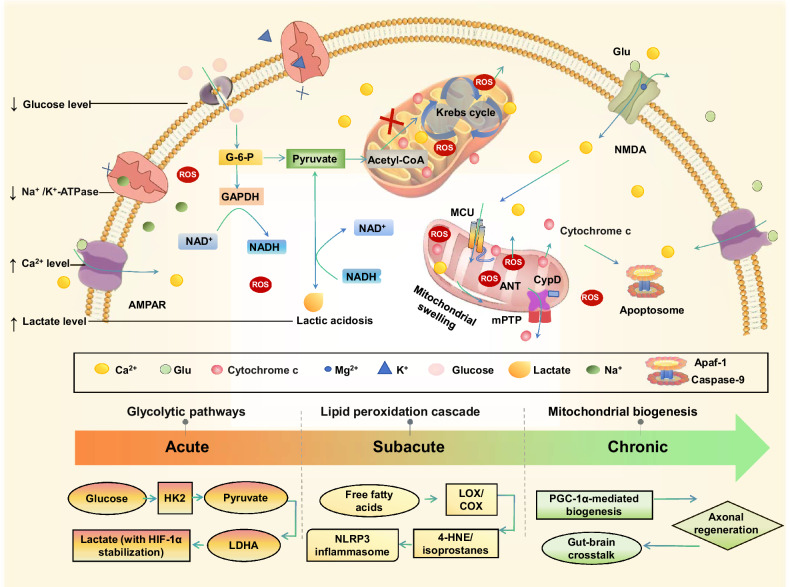
Temporal dynamics of stroke-induced metabolic and ion channel dysregulation across neuronal and glial cells. Acute bioenergetic failure, subacute inflammatory responses, and chronic recovery following ischemic stroke: in the acute phase, neurons exhibit rapid metabolic collapse, including impaired glycolysis (glucose-to-pyruvate conversion) and mitochondrial dysfunction, culminating in severe ATP depletion. Dysregulated ion flux drives excitotoxicity: delayed voltage-gated Na^+^ channel inactivation, impaired K^+^ channel repolarization, and pathological Ca^2+^ overload via NMDA receptors/VGCCs. Systemic contributors (e.g., BBB leakage) amplify injury, while PARP-1 hyperactivation depletes NAD^+^, worsening energy failure. In the subacute Phase (days), glial metabolic-ionic crosstalk emerges. Astrocytes shift toward compensatory glycolysis and lipid droplet accumulation, reducing glutamate uptake due to suppressed GLT-1 transporters. Microglia adopt a pro-inflammatory phenotype, releasing cytokines (IL-1β, TNF-α) and altering K^+^ (KCNJ2) and Ca^2+^ (TRPM2) channels to sustain activation. Free fatty acid accumulation and NAD^+^/SIRT1 depletion exacerbate mitochondrial damage, fueling NLRP3 inflammasome formation and eicosanoid production (LOX/COX; 4-HNE/isoprostanes). In the chronic phase, persistent metabolic stress triggers mitochondrial biogenesis via PGC-1α, while gut-brain crosstalk may modulate axonal regeneration. AMPK activation (via ATP ↓ ) promotes glucose uptake through TBC1D1 phosphorylation, offering potential recovery pathways amid sustained neuroinflammation. GLT-1 glutamate transporter 1, NMDA *N*-methyl-*D*-aspartate receptor, TRPM2 transient receptor potential melastatin 2, VGCC voltage-gated calcium channel, G-6-P glucose-6-phosphate, acetyl-CoA acetyl coenzyme A, GAPDH glyceraldehyde-3-phosphate dehydrogenase, NADH nicotinamide adenine dinucleotide (reduced form), AMPAR α-amino-3-hydroxy-5-methyl-4-isoxazolepropionic acid receptor, MCU mitochondrial calcium uniporter, CypD cyclophilin D, ANT adenine nucleotide translocator, mPTP mitochondrial permeability transition pore, Apaf-1 apoptotic peptidase activating factor 1, Caspase-9 cysteinyl aspartate-specific proteinase 9. Partially created by Biorender.com.

These interconnected pathways operate beyond traditional barriers. Plasma protein uptake into brain parenchyma occurs constitutively via receptor-mediated transcytosis [78] (Fig. 1), establishing the gliovascular interface as a conduit for bidirectional signal exchange [79]. Post-stroke BBB hyperpermeability accelerates peripheral immune cell infiltration, amplifying metabolic stress through reactive oxygen species and cytokine storms. Anatomical connections, including autonomic innervation of the heart/liver/gut [80, 81], glymphatic clearance pathways [82], and direct vascular channels permitting immune cell trafficking from calvaria to meninges [83, 84], facilitate central-peripheral communication. These functional and anatomical pathways enable the transmission of neural, cellular, and molecular signals, creating extensive crosstalk between the neurovascular unit and systemic biology. Understanding these interactions linking systemic metabolism, immune responses, and potentially even the microbiome could unveil mechanisms of stroke pathophysiology, identify diagnostic biomarkers, and point to therapeutic targets, thereby bridging experimental research with clinical applications [10–12, 85–102] (Table 3).

**Table 3:** Candidate MRIS biomarkers and therapeutic agents in clinical trials.

Biomarker/target	Source/agent	Pathway	MRIS mechanism	Trial phase	Function/impact	Challenges
β-hydroxybutyrate (BHB)	Plasma	Ketogenesis	Alternative energy substrate during glucose deprivation [10]	NA	Correlates with improved neurological outcomes [11]	Variability due to fasting; sampling standardization needed [12]
CSF itaconate	CSF	NLRP3 signaling	SDH inhibition → dampens NLRP3 activation [101]	NA	Reduced levels link to exacerbated neuroinflammation [100]	Invasive sampling; analyte instability [102]
Lactate	CSF	Anaerobic glycolysis	Byproduct of glycolytic stress [102]	NA	Predicts infarct expansion/poor outcomes [97]	Non-specific elevation in TBI/sepsis
Malondialdehyde	Serum	Lipid peroxidation	Ferroptosis marker via lipid peroxidation [85]	NA	Correlates with stroke severity/infarct volume [86]	Plasma instability; rapid processing required
Mitochondrial DNA (mtDNA)	Plasma	TLR9/ stimulator of interferon genes signaling	Mitochondrial damage → neuroinflammation amplification [87]	NA	Predicts worse functional recovery [88]	Quantification variability; nuclear DNA contamination [88]
COX-2 inhibitors	Celecoxib	Arachidonic acid metabolism	Prostaglandin synthesis inhibition	Phase III	Reduced IL-6/TNF-α but ↑ cardiovascular risk [89]	Narrow window; hemorrhagic risk
DGAT1 inhibitors	Pradigastat	Lipid droplet formation	Triglyceride synthesis blockade [90]	Phase II	Reduced neuronal lipid peroxidation [99]	GI side effects limit tolerability [98]
Edaravone	NA	ROS scavenging	Free radical neutralization	Approved (post-hoc)	Modest NIHSS improvement in subgroups [91]	Short half-life; requires infusion
MitoQ	NA	Mitochondrial OXPHOS	Targeted antioxidant; ↑ OXPHOS efficiency [92]	Preclinical	30% infarct reduction in rodents [93]	Limited BBB penetration
Natalizumab	NA	Immune cell trafficking	α4-integrin inhibition → blocks infiltration [94]	Phase II	Reduced microglial activation/IL-1β [95]	Opportunistic infection risk (e.g., PML) [96]
AMPK	Metformin	Energy sensing	AMPK activation → glycolysis inhibition	Phase II (NCT04524702)	Reduces lactate-driven acidosis	Hypoglycemia risk in diabetics
NAD^+^ salvage	Nicotinamide Riboside	NAD^+^ biosynthesis	Boosts NAD^+^ → activates SIRT1/PARP-1	Phase III (NCT05143996)	Improves cognitive recovery	Limited efficacy in aged mitochondria
mTOR	Rapamycin analogs	Lipid synthesis regulation	mTORC1 inhibition → ↓ lipid synthesis	Preclinical	Attenuates astrocytic lipid droplet accumulation	Immunosuppression side effects
SIRT1	SRT2104	Mitochondrial resilience	SIRT1 activation → enhances PGC-1α	Phase I (NCT03886186)	Promotes mitochondrial biogenesis	Tissue-specific delivery challenges

### Neuronal energetics

Ischemic stroke triggers a cascade of excitotoxic and metabolic failures that critically disrupt neuronal energetics. Central to this cascade is glutamate-induced hyperactivation of *N*-methyl-*D*-aspartate receptors (NMDA receptors), driving pathological calcium influx that overwhelms mitochondrial buffering capacity [103] (Fig. 1). This ion imbalance uncouples oxidative phosphorylation, depleting ATP and impairing Na^+^/K^+^-ATPase function. Consequently, membrane depolarization intensifies, facilitating further glutamate release and establishing a self-perpetuating cycle of excitotoxicity [104]. Simultaneously, delayed sodium channel inactivation and impaired potassium repolarization exacerbate calcium overload, accelerating metabolic collapse.

Mitochondrial dysfunction amplifies reactive oxygen species (ROS) production, inducing DNA damage and hyperactivating PARP-1 [105]. PARP-1 hyperactivation depletes cytosolic NAD^+^, a central redox cofactor essential for glycolysis and TCA cycle function, precipitating bioenergetic failure. This NAD^+^ deficit directly impairs sirtuin activity (SIRT), compromising their regulation of mitochondrial biogenesis via PGC-1α, antioxidant defense through FOXO transcription factors, and metabolic adaptation via HIF-1α modulation [106, 107]. Although neurons activate compensatory NAD^+^ salvage through nicotinamide phosphoribosyltransferase (NAMPT) [108], this pathway fails to offset PARP-1-driven NAD^+^ catabolism during stroke [106], resulting in sustained sirtuin suppression and disrupted mitochondrial dynamics. Consequently, the NAD^+^/NADH imbalance propagates metabolic inflexibility, impairing neuron-glia metabolic crosstalk while dysregulating the α-amino-β-carboxymuconate-ε-semialdehyde decarboxylase (ACMSD)/kynurenine pathway, a critical alternate NAD^+^ biosynthesis route [109, 110]. Critically, acute AMPK activation during ATP depletion exacerbates glycolytic overdrive and FAO, while chronic mTORC1 signaling in surviving neurons drives maladaptive lipid synthesis that fuels ferroptosis [111]. Metabolic disruption also extends to thrombotic mechanisms: platelet activation requires a glycolytic ATP surge mediated by HK2 and 6-phosphofructo-2-kinase/fructose-2,6-biphosphatase 3 (PFKFB3), while hyperglycemia stiffens clots via advanced glycation end-product (AGE) crosslinking. As a result, thrombectomy alone is insufficient if these metabolic drivers persist, underscoring the need for metabolic co-interventions alongside mechanical reperfusion.

Preclinical studies have suggested that inhibiting PARP-1 or supplementing NAD^+^ could offer neuroprotective benefits [112]. However, translating these strategies into clinical practice remains challenging due to potential off-target effects and limitations in crossing the BBB. Additionally, emerging evidence suggests that NAD^+^ precursors, such as nicotinamide riboside, may help alleviate post-stroke cognitive deficits by restoring synaptic plasticity [106, 113]. The complex interplay among glutamate excitotoxicity, mitochondrial collapse, and NAD^+^ dysregulation characterizes the disruption of neuronal energetics in strokes. Therapeutic approaches targeting these pathways with glutamate receptor antagonists, PARP-1 inhibitors, or NAD^+^ enhancers are promising for breaking the cycle of metabolic failure. However, the therapeutic windows for effective intervention remain narrow, emphasizing the need for refined strategies that also address thrombo-metabolic convergence.

### Astrocytic metabolic switch

Astrocytes exemplify MRIS’s spatiotemporal duality: their early glycolytic support later evolves into lipid-driven neuroinflammation. This shift directly impacts microglial behavior, where succinate accumulation triggers inflammatory cascades, highlighting how MRIS propagates injury across cell types [114]. Acute ischemia initiates glycogenolysis in astrocytes, releasing glucose-6-phosphate that fuels glycolysis and lactate production [97, 102]. This astrocyte-neuron lactate shuttle initially sustains neuronal ATP synthesis; however, as lactate accumulates, it leads to acidosis and an increase in ROS, ultimately becoming detrimental to neuronal health [115] (Fig. 1). Prolonged glycolysis depletes astrocytic glycogen reserves, prompting a metabolic shift toward FAO and the accumulation of lipid droplets (LDs) [116]. Once regarded as inert storage depots, LDs are now recognized as active immunometabolic hubs that sequester toxic lipids and release pro-inflammatory mediators, including prostaglandin E2 (PGE2) [117, 118].

The metabolic landscape of astrocytes is further complicated by HIF-1α, which upregulates pyruvate dehydrogenase kinase (PDK) [6]. This action diverts pyruvate from the TCA cycle, funneling it instead toward lactate production. While this pseudo-hypoxic state may provide short-term adaptive benefits, it ultimately impairs mitochondrial respiration and heightens oxidative stress [119]. Recent advances in single-cell RNA sequencing have identified subpopulations of “lipid-laden astrocytes” that express LD-associated proteins, such as PLIN2, alongside pro-inflammatory cytokines [120]. Notably, the presence of these astrocyte subpopulations correlates with poorer outcomes in murine models of stroke. The dual nature of astrocytic metabolism to balance neuroprotective lactate shuttling with pro-inflammatory lipid signaling underscores the importance of temporally precise therapeutic interventions. Pharmacological inhibition of LDs formation through DGAT inhibitors or modulation of HIF-1α activity may mitigate chronic neuroinflammation without compromising acute energetic support [90, 98, 99].

### Microvascular metabolism

The cerebral microvasculature is both a victim and amplifier of metabolic dysfunction in stroke. Endothelial cells, reliant on glycolysis under normoxia, switch to FAO during ischemia, driven by HIF-2α upregulation of carnitine palmitoyltransferase 1A (CPT1A) [121]. While FAO sustains ATP production, it generates excess acetyl-CoA, inhibiting pyruvate dehydrogenase and exacerbating lactate accumulation. This metabolic shift also reduces nitric oxide (NO) bioavailability, impairing vasodilation and promoting thrombus formation.

Pericytes, contractile mural cells regulating capillary tone, exhibit HIF-1α-mediated RhoA (Ras homolog gene family, member A)/Rho-associated protein kinase (ROCK) pathway activation, leading to sustained contraction and capillary “no-reflow” post-recanalization [122]. Pericyte contraction, initially hemodynamic, is fueled by astrocyte-derived lactate activating acid-sensing ion channels (ASICs), positioning metabolic modulation via LDHA inhibition as a potential strategy to rescue perfusion [123]. Furthermore, pericyte-derived inflammatory mediators such as IL-6 recruit neutrophils, exacerbating BBB disruption. Therapeutic strategies targeting microvascular metabolism include CPT1A inhibitors to normalize endothelial bioenergetics and ROCK inhibitors like fasudil to alleviate pericyte constriction [124, 125]. Notably, HIF stabilization agents like roxadustat show dual benefits, enhancing endothelial cells' resilience while suppressing pericyte hyperactivation in preclinical models [126]. However, balancing these interventions to avoid off-target effects on angiogenesis remains critical. MRIS does not explain why vascular occlusion occurs but reveals how its consequences propagate systemically. Stroke remains etiologically heterogeneous, ranging from cardioembolic to lacunar causes, with MRIS serving as a common downstream amplifier of injury.

### MRIS and vascular disease with metabolic sequelae: shared pathways and amplified injury

Pre-existing vascular diseases accompanied by metabolic sequelae profoundly exacerbate MRIS by hijacking its core pathways, creating vicious cycles that amplify ischemic injury. In metabolic dysfunction-associated steatotic liver disease, impaired hepatic ketogenesis reduces circulating β-hydroxybutyrate (BHB), depriving neurons of a neuroprotective fuel and worsening energy failure. Rodents with hepatic steatosis develop 40% larger infarcts due to defective ketogenesis, a deficit reversible with BHB supplementation [127, 128]. Obesity primes adipose NLRP3 inflammasomes, elevating systemic free fatty acids (FFAs) and IL-1β that intensify cerebral lipid peroxidation. Clinically, body mass index (BMI > 30) correlates with 2.5× higher cerebrospinal fluid (CSF) IL-1β and larger peri-infarct lipid peroxide deposits [19].

Diabetic hyperglycemia triggers paradoxical glycolysis suppression despite energy deficits, while microglial advanced glycation end products (RAGE) receptors, a dual insult exacerbating acidosis and neuroinflammation, are evidenced by diabetics exhibiting 3× higher lactate/penumbra volume ratios than non-diabetics [46, 129]. Hypertension, conversely, upregulates endothelial FAO, reducing nitric oxide bioavailability and capillary perfusion, explaining why hypertensives gain 50% less reperfusion benefit post-thrombectomy [122, 130, 131]. These comorbidities converge on mitochondrial ROS overproduction and NLRP3-driven neuroinflammation. Diabetic hyperglycemia further exacerbates this by suppressing astrocytic pentose phosphate pathway flux, which depletes nicotinamide adenine dinucleotide phosphate (NADPH) reserves [132, 133], a mechanism that mirrors defects in diabetic neuropathy where impaired redox defense amplifies oxidative injury. This metabolic synergy is illustrated by the case of a 65-year-old diabetic woman with a BMI of 34 and apolipoprotein E 4 (APOE4) genotype, presenting with cardioembolic stroke [47]. Her personalized MRIS intervention was tailored to address specific vulnerabilities identified through stratification, including elevated TMAO levels indicative of gut dysbiosis, low β-hydroxybutyrate reflecting impaired diabetic ketogenesis, and high low-density lipoprotein linked to an APOE4-driven lipid efflux defect [120]. Consequently, metformin was withheld due to the risk of lactic acidosis, and ketogenic therapy was avoided, given APOE4-associated lipid sensitivity [120, 132]. Targeted therapeutics included a TMA lyase inhibitor, Akkermansia probiotic supplementation, and low-dose rosuvastatin. This phenotype-specific modulation of MRIS resulted in a 45% reduction in infarct growth at 72 h (magnetic resonance imaging-diffusion weighted imaging, DWI) compared to standard care, validating the approach [13, 28]. Importantly, this convergence of comorbidities helps explain broader divergences in stroke outcomes: the same MRIS pathway, such as the glycolytic surge that drives lipid peroxidation in obesity, also accelerates endothelial dysfunction in hypertension, underscoring the critical need for precision stratification and tailored interventions.

## Metabolic reprogramming aggravating neuroinflammation post-stroke

### Lactate accumulation and HIF-1α signaling

During ischemic stroke, hypoxia and glucose deprivation compel cells to transition from oxidative phosphorylation to anaerobic glycolysis [115, 133, 134]. While glycolysis provides a rapid ATP source, its low ATP yield drives intracellular lactate accumulation. Critically, lactate exhibits concentration-dependent duality: at <5 mM, it stabilizes HIF-1α to promote anti-inflammatory microglial M2 polarization and serves as an alternative neuronal fuel; however, at >5 mM, it induces acidosis, inactivates pH-sensitive enzymes, and activates apoptotic signaling pathways [135] (Fig. 1)

Furthermore, lactate stabilizes HIF-1α, which in turn promotes the expression of vascular endothelial growth factor [135]. This action disrupts the integrity of the BBB, increasing its permeability. In microglial BV2 cells subjected to oxygen-glucose deprivation, lactate has been shown to upregulate the chemokine Chemokine (C-C motif) ligand 7 (CCL7) through HIF-1α signaling. Post-reperfusion, regions surrounding the infarct show elevated CCL7 expression, and inhibition of HIF-1α reverses lactate-induced neuroinflammation and infarct expansion [136]. The administration of recombinant CCL7 counteracts the neuroprotective effects of lactate, highlighting the critical role of the HIF-1α–CCL7 axis in modulating neuroinflammatory responses following stroke. Microglial pro-inflammatory polarization is further sustained by altered potassium (KCNJ2, potassium inwardly rectifying channel subfamily J member 2) and calcium (TRPM2, transient receptor potential melastatin 2) channel activity, which regulate cytokine release and immune activation.

### Ion pump dysfunction and excitotoxicity

The brain’s dependence on oxidative phosphorylation renders it susceptible to energy failures during ischemia. The depletion of ATP impairs the maintenance of ion gradients, resulting in membrane depolarization in both neurons and glial cells [137] (Fig. 1). This depolarization activates voltage-gated calcium channels, leading to excessive glutamate release into the extracellular space. Concurrently, the failure of energy-dependent glutamate reuptake mechanisms exacerbates extracellular glutamate levels.

The resulting glutamate overstimulation of NMDA and metabotropic glutamate receptors (mGluRs) further complicates the situation, whereby mGluR activation via phospholipase C (PLC) and inositol trisphosphate (IP_3_) signaling prompts Ca^2+^ overload [138]. Moreover, α-amino-3-hydroxy-5-methyl-4-isoxazolepropionic acid (AMPA) receptor-mediated Na^+^/Cl^−^ influx further disturbs osmotic balance, contributing to cytotoxic edema, a process blocked by AMPA antagonists (e.g., perampanel) in rodent models [139, 140]. This edema is detrimental, compromising perfusion in peri-infarct regions, elevating intracranial pressure, and increasing the risk of herniation, an important factor contributing to early mortality. Imaging modalities such as magnetic resonance imaging and computed tomography are essential for monitoring edema during acute stroke management.

### Amino acid metabolic reprogramming post-stroke

Ischemic stroke incites profound alterations in amino acid metabolism. The excessive accumulation of excitatory neurotransmitters, particularly glutamate, drives excitotoxicity, while metabolites such as arginine regulate inflammatory responses through NO synthesis and urea cycle modulation. Recent studies position amino acid metabolism as an increasingly important therapeutic target in stroke [141–143] (Table S2).

**Fig. 2:**
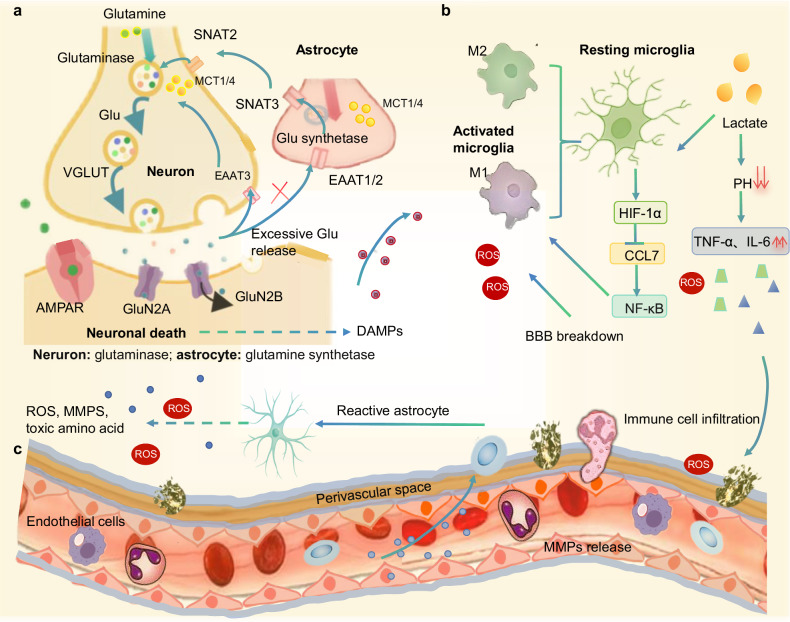
Metabolic reprogramming and neuroimmune crosstalk in ischemic stroke. **a** Under normal conditions, the glutamate–glutamine cycle maintains synaptic balance. Neurons import glutamine via SNAT3 transporters, converting it to glutamate (Glu) via glutaminase (GLS). Synaptic Glu release occurs through vesicular glutamate transporters (VGLUTs), with partial neuronal reuptake via EAAT3. Astrocytes uptake excess Glu via EAAT1/2, convert it to glutamine via glutamine synthetase (GS), and shuttle glutamine back to neurons via SNAT2/3. Lactate shuttling via MCT4 (astrocyte export) and MCT1 (neuronal import) fuels neuronal metabolism, supporting glutamate cycling. **b** Lactate-driven microglial polarization: Elevated lactate (transported via MCT1/4) lowers extracellular pH, stabilizing HIF-1α in microglia. HIF-1α suppresses chemokine CCL7, inhibiting NF-κB activation. This reduces pro-inflammatory cytokines (TNF-α, IL-6) and promotes a shift to anti-inflammatory M2 phenotypes. **c** Secondary injury cascade: BBB disruption facilitates immune cell infiltration and lactate accumulation. Reactive astrocytes exacerbate tissue damage by releasing matrix metalloproteinases (MMPs), ROS, and neurotoxic factors (excess glutamate). Neuronal death releases damage-associated molecular patterns (DAMPs) that perpetuate microglial activation and ROS production. Systemic contributors amplify this cascade: hepatic ketogenesis modulates cerebral energetics, gut-derived microbial metabolites (SCFAs/TMAO) traverse the compromised BBB to influence neuroinflammation, and sympathetic activation of bone marrow drives neutrophil egress. Bidirectional interactions (arrowheads) between metabolic dysfunction, ion imbalance, and neuroinflammation create a self-reinforcing cycle of injury, further intensified by peripheral immune cells infiltrating through the compromised BBB. Partially created by Biorender.com.

As the principal excitatory neurotransmitter, glutamate activates AMPA, NMDA, and kainate receptors. During ischemia, overstimulation of NMDA receptors by glutamate and glycine triggers pathological calcium influx, leading to mitochondrial calcium overload and subsequent mitochondrial permeability transition pore (mPTP) opening, further initiating apoptotic cascades [144] (Fig. 2a). The accumulation of extracellular glutamate exacerbates oxidative stress and neuronal death, hallmarks of ischemic injury as well as neurodegenerative diseases. Neuronal death releases damage-associated molecular patterns such as high mobility group box proteins (HMGB1), which perpetuate microglial activation and ROS production [145]. Bidirectional interactions between metabolic dysfunction (e.g., lactate-driven acidosis) and neuroinflammation (e.g., cytokine storms) create a self-reinforcing injury loop, amplified by peripheral immune infiltration through the compromised BBB.

Interestingly, the small-molecule amino acid (AA)-147, which activates the unfolded protein response transcription factor activating transcription factor 6 (ATF6), protects against glutamate-induced toxicity by covalently modifying KEAP1, thereby activating NRF2-dependent antioxidant pathways [146]. However, sustained NMDA receptor activation can reverse the cystine/glutamate antiporter transporter’s action, leading to intracellular glutathione depletion and amplifying oxidative damage [147–149].

### Neuron–astrocyte metabolic crosstalk

In the healthy brain, neurons and astrocytes sustain a delicate metabolic symbiosis through the glutamate–glutamine cycle, a lifeline where neurons convert astrocyte-derived glutamine into glutamate for neurotransmission, while astrocytes rapidly clear synaptic glutamate via excitatory amino acid transporters (EAAT1/2), detoxify it to glutamine via glutamine synthetase, and return this precursor to neurons to sustain synaptic function [150] (Fig. 2a). This elegant partnership unravels catastrophically during ischemic stroke. Surging neuronal glutamate release overwhelms astrocytic uptake capacity, flooding the extracellular space with excitotoxic glutamate. Compounded by astrocytic EAAT dysfunction, the cycle collapses, triggering a metabolic emergency. Under physiological conditions, neurons import glutamine via sodium-coupled neutral amino acid transporter 3 (SNAT3), converting it to glutamate via glutaminase for synaptic release through vesicular glutamate transporters (VGLUTs). Astrocytes clear excess glutamate via EAAT, convert it to glutamine via glutamine synthetase, and shuttle glutamine back [151] via SNAT2/3. Ischemic stroke disrupts this cycle: SNAT dysfunction impairs glutamine supply, while VGLUT hyperactivity and EAAT failure exacerbate extracellular glutamate accumulation. Astrocytes, now forced into anaerobic glycolysis, overproduce lactate [152], a metabolite with context-dependent roles. Through MCT-mediated transport, elevated lactate lowers extracellular pH, stabilizing HIF-1α in microglia. This HIF-1α activation suppresses chemokine CCL7, inhibiting NF-κB and reducing pro-inflammatory cytokines such as tumor necrosis factor-alpha (TNF-α) and IL-6, thereby promoting anti-inflammatory M2 polarization. Paradoxically, in early ischemia, lactate overabundance fuels a pro-inflammatory M1-dominant milieu [153]. These activated microglia unleash ROS and IL-1β, compromising BBB integrity and recruiting peripheral immune cells (Fig. 2b).

Amid this chaos, astrocytes diverge into functionally opposing phenotypes: cytotoxic A1-reactive astrocytes secrete MMP-9 and ROS, actively eroding the neurovascular unit, while reparative A2 astrocytes deploy Wnt/β-catenin signaling and TLR4-mediated neurotrophin release to facilitate synaptic rewiring and functional reorganization [154, 155]. The astrocyte-neuron lactate shuttle, once a routine energy exchange, becomes a critical survival mechanism. Astrocyte-derived lactate, shuttled via MCT, sustains neuronal mitochondrial respiration during glucose deprivation, while oligodendrocytes extend this metabolic lifeline to axons, preserving ionic gradients and axonal integrity [152]. However, this adaptation is perilously double-edged. Lactate accumulation acidifies the microenvironment, exacerbating ion dyshomeostasis and attracting neutrophil extracellular traps, web-like DNA structures that entangle MMP-9, amplifying BBB leakage [156] (Fig. 2c). Regulatory T cells counterbalance this damage, modulating MMP-9 activity to stabilize the barrier, but their delayed recruitment often cedes ground to early inflammatory cascades.

The ischemic brain thus embodies a metabolic paradox: accelerated glycolysis provides immediate ATP but promotes long-term injury through acidosis and oxidative stress; lactate shifts from neuroprotective fuel to pro-inflammatory mediator as concentrations exceed 5 mM; and astrocytes transition from neurosupportive roles of sustaining neuronal energetics via lactate shuttling to neurotoxic contributors through chronic lipid droplet accumulation that exacerbates neuroinflammation. Reconciling these roles demands precision, targeting the timing and spatial distribution of lactate flux, restoring glutamate–glutamine cycle fidelity, or harnessing A2 astrocyte plasticity. As we dissect this metabolic tango, the goal is clear: to transform the post-stroke brain from a battlefield of competing signals into a scaffold for rebirth, where neuron–astrocyte crosstalk is not merely salvaged but strategically rewired.

### Mitochondrial dysfunction and oxidative stress

**Fig. 3:**
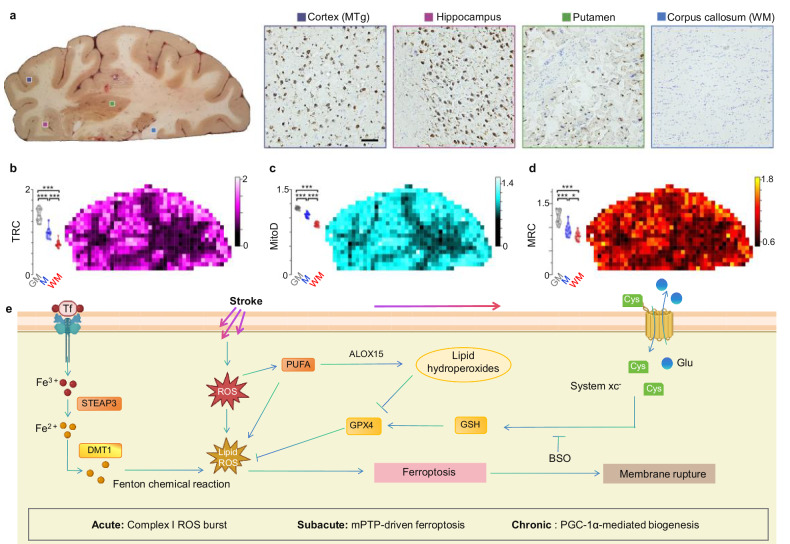
Mitochondrial dysfunction and cell-type-specific metabolic reprogramming in ischemic stroke. **a** Regional mitochondrial heterogeneity. **b**–**d** Ischemic vulnerability. **b** Tissue respiratory control (TRC) maps and **c** Mitochondrial Respiratory Capacity (MRC) maps reveal reduced oxidative phosphorylation (OXPHOS) efficiency in stroke-affected regions, correlating with selective neuronal loss in metabolically active gray matter (GM). Impaired OXPHOS (↓MRC/TRC) exacerbates ROS overproduction and calcium overload, driving ferroptosis. **d** Mitochondrial Density (MitoD) is 50% higher in GM than in white matter, explaining GM’s susceptibility to energy failure during ischemia. Reprinted from ref[49]. Springer Nature Limited. **e** Schematic of the ferroptosis pathway in high-OXPHOS neurons: ROS burst → Fe^2+^-dependent PUFA peroxidation → ALOX15 activation → GPX4 inhibition via glutathione depletion → irreversible membrane rupture. Temporal mitochondrial dysfunction: In the acute phase, complex I-driven ROS burst initiates injury. In the subacute phase, mPTP opening triggers Fe^2+^ release from storage proteins (Tf/STEAP3/DMT1), driving ferroptosis. In the chronic phase, Compensatory PGC-1α-mediated biogenesis promotes recovery. Tf transferrin, STEAP3 six-transmembrane epithelial antigen of prostate 3, DMT1 divalent metal transporter 1, PUFA polyunsaturated fatty acid, GPX4 glutathione peroxidase 4, BSO buthionine sulfoximine, mPTP mitochondrial permeability transition pore.

Mitochondria are central to ATP production through oxidative phosphorylation, but they become structurally and functionally compromised during ischemia. The electron transport chain, comprising complexes I to V, ubiquinone, and cytochrome c, generates a proton gradient that drives ATP synthesis [157]. In the face of ischemic conditions, oxygen scarcity halts electron transport chain activity, leading to reduced ATP output and increased ROS production at the ubiquinone-cytochrome c site (Fig. 3a–d). Regional mitochondrial heterogeneity dictates ischemic vulnerability: Gray matter, with 50% higher mitochondrial density (MitoD) than white matter, exhibits heightened susceptibility to energy failure during ischemia [49] (Box 1). Impaired mitochondrial respiratory capacity in high oxidative phosphorylation (OXPHOS) neurons reduces ATP synthesis, generating superoxide radicals that overwhelm superoxide dismutase defenses [158]. This acute complex I-driven ROS burst initiates injury through Fe^2+^-dependent peroxidation of polyunsaturated fatty acids in mitochondrial membranes via Fenton chemistry [159]. During the subacute phase, mitochondrial permeability transition pore opening triggers Fe^2+^ release from storage proteins, transferrin/six-transmembrane epithelial antigen of prostate 3 (STEAP3)/divalent metal transporter 1 (DMT1), driving ferroptosis. The execution pathway involves: ROS activating lipoxygenases (ALOX)-15 via calcium-mediated phospholipase A_2_ (releasing arachidonic acid) [160]; lipid peroxide accumulation depleting glutathione, the essential GPX4 cofactor; glutathione deficiency directly inactivating GPX4, disabling phospholipid peroxide reduction; and culminating in irreversible membrane rupture [144, 148]. Concurrently, cytosolic calcium overload from mitochondrial calcium uniporter inactivation [161] accelerates this cascade. In chronic phases, compensatory PGC-1α-mediated mitochondrial biogenesis promotes recovery [162]. Experimental evidence confirms this mechanism: GPX4-knockout mice show 3-fold larger infarcts with lipid peroxide accumulation in high-OXPHOS regions, while ferroptosis inhibitors reduce neuronal loss by 60% in middle cerebral artery occlusion (MCAO) models [163, 164] (Fig. 3e). Experimental evidence includes: GPX4-knockout mice show 3-fold larger infarcts with lipid peroxide accumulation in high-OXPHOS regions, while ferroptosis inhibitors (e.g., ferrostatin-1) reduce neuronal loss by 60% in MCAO models [163, 165].

The process of reperfusion intensifies ROS generation, overwhelming the brain’s antioxidant defenses, such as superoxide dismutase and glutathione peroxidase. This oxidative stress leads to lipid peroxidation, protein oxidation, and mitochondrial DNA damage. Mitochondrial pyruvate oxidation generates citrate, which is exported to the cytoplasm via ATP-citrate lyase for acetyl-CoA production, fueling lipid synthesis and protein acetylation [166]. Accumulation of saturated fatty acids (SFAs) such as palmitate [C16:0], and stearate [C18:0] disrupts mitochondrial dynamics, impairing motility and ATP synthesis while promoting ROS generation [167]. ROS are also responsible for disrupting mitochondrial calcium homeostasis, a process mediated by voltage-dependent anion channels and the mitochondrial calcium uniporter [168]. Elevated calcium levels within the mitochondrial matrix can depolarize the mitochondrial membrane potential (ΔΨm), resulting in the opening of the mPTP [169]. The opening of mPTP, which is regulated by adenine nucleotide translocase and cyclophilin D, facilitates the release of pro-apoptotic factors, including cytochrome c and apoptosis-inducing factors, into the cytosol. Cytochrome c activates caspase-9 through apoptosome formation, culminating in the activation of caspase-3 and subsequent PARP-mediated DNA damage. These cascades dramatically enhance neuronal loss through apoptosis and necroptosis.

Box 1 Metabolic heterogeneity deciphers behavioral resilience in stroke recovery Single-cell and spatial omics technologies from RNA sequencing to spatial metabolomics have unmasked how metabolic reprogramming shapes neurobehavioral trajectories in ischemic stroke. These tools reveal that cellular metabolic states are not mere biochemical footnotes but determinants of cognitive recovery, motor function, and emotional adaptation. Lipid-laden astrocytes, clustered in peri-infarct regions, correlate with delayed rehabilitation progress in preclinical models, their lipid droplets creating neurotoxic microenvironments that impair synaptic repair. Conversely, the presence of lactate-producing microglia subsets is linked to regions associated with preserved executive function, indicating that metabolic crosstalk might play a role in resilience to post-stroke depression. Spatial mapping further exposes stark contrasts: gray matter neurons, vulnerable to excitotoxicity due to high oxidative demand, map to domains of memory impairment, while glycolytic oligodendrocyte precursors in white matter sustain axonal integrity critical for motor recovery. Clinically, these insights are driving trials of metabolic precision therapies, such as DGAT1 inhibitors to dismantle astrocytic lipid toxicity, or circadian-timed HIF-1α modulators to rebalance microglial polarization tailored for patient-specific metabolic subphenotypes identified via omics profiling [49].

### Lipid metabolic reprogramming and axonal regeneration

**Fig. 4:**
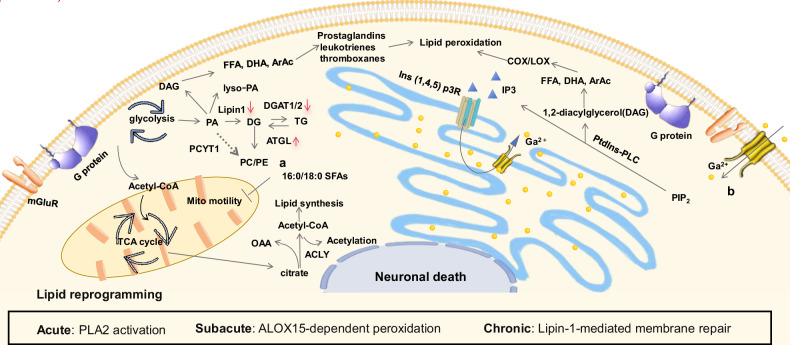
Lipid metabolic reprogramming and oxidative injury in ischemic neurons. **a** Energy metabolism and mitochondrial dysfunction. Glycolysis converts glucose to pyruvate, which enters mitochondria via pyruvate dehydrogenase (PDH) to form acetyl-CoA for the TCA cycle. Citrate is exported to the cytoplasm and processed by ATP-citrate lyase into acetyl-CoA for lipid synthesis and protein acetylation. Accumulation of saturated fatty acids (SFAs; e.g., palmitate [16:0] and stearate [18:0]) disrupts mitochondrial dynamics and motility. In response to excess lipid accumulation and oxidative stress, astrocytes initiate mTOR-dependent lipid droplet formation, mediated by diacylglycerol acyltransferase 1 (DGAT1) and PLIN2, which sequesters toxic lipids and buffers oxidative injury. **b** Glutamate receptor signaling and calcium dysregulation: Ischemia triggers excessive glutamate release, activating metabotropic glutamate receptors (mGluR) and G-protein-coupled pathways. This stimulates phosphatidylinositol-specific PLC, cleaving phosphatidylinositol 4,5-bisphosphate (PIP_2_) into inositol trisphosphate (IP_3_) and diacylglycerol (DAG). IP_3_ binds to its receptor (InsP_3_R) on the endoplasmic reticulum (ER), releasing stored calcium (Ca^2+^), which exacerbates intracellular Ca^2+^ overload. DAG and FFAs, including docosahexaenoic acid (DHA) and arachidonic acid (ArAc), are metabolized by cyclooxygenase (COX) and lipoxygenase (LOX) into pro-inflammatory mediators (prostaglandins, leukotrienes, thromboxanes). Concurrently, enzymes such as DGAT1/2, adipose-triglyceride lipase (ATGL), phosphate cytidylyltransferase 1 (PCYT1), and Lipin-1 drive aberrant lipid turnover, promoting lipid peroxidation. Lipid reprogramming across stroke phases: In the acute phase, activation of phospholipase A2 (PLA2) promotes the release of FFAs and lysophospholipids, triggering rapid membrane destabilization and inflammatory lipid signaling. In the subacute phase, enhanced ALOX15-dependent lipid peroxidation of polyunsaturated fatty acids leads to ferroptotic neuronal death. In the chronic phase, Lipin-1-mediated membrane repair supports phospholipid remodeling and recovery of membrane integrity. G protein Guanine nucleotide binding protein, Ins(1,4,5)p3R inositol 1,4,5-trisphosphate receptor, PtdIns-PLC Phosphatidylinositol-specific phospholipase C, PCYT1 phosphocholine cytidylyltransferase 1, DATGL adipose-triglyceride lipase, ACLY ATP-citrate lyase, IP3 Inositol 1,4,5-trisphosphate, PIP_2_ phosphatidylinositol 4,5-bisphosphate, FFA free fatty acid, DHA docosahexaenoic acid, ArAc arachidonic acid, PA phosphatidic acid, lyso-PA lysophosphatidic acid, DG diacylglycerol, TG triacylglycerol, PC/PE phosphatidylcholine/phosphatidylethanolamine, 16:0/18:0 SFAs saturated fatty acids with 16 or 18 carbon atoms.

Following ischemic injury, neurons undergo significant lipid metabolic reprogramming to support membrane repair and axonal growth [170]. This process centers on enhanced phospholipid synthesis, where Lipin-1 deficiency promotes phosphatidic acid accumulation, activating phosphate cytidylyltransferase 1 (PCYT1) to drive phosphatidylcholine production [171], a critical component for reconstructing damaged membranes. Concurrently, triglyceride hydrolysis mediated by adipose-triglyceride lipase (ATGL) and DDHD2 liberates fatty acids from lipid droplets, providing substrates for membrane biogenesis [172]. The fate of these fatty acids diverges critically: long-chain SFAs disrupt mitochondrial motility and impair axonal transport, while short-chain fatty acids (C12:0/C14:0) preserve mitochondrial integrity and support regeneration [173] (Fig. 4a).

Ischemic glutamate receptor hyperactivation stimulates phosphatidylinositol-specific PLC, cleaving PIP_2_ into IP_3_ and diacylglycerol (DAG). IP_3_ then triggers calcium release from endoplasmic reticulum stores, exacerbating intracellular calcium overload. DAG and free fatty acids (e.g., arachidonic acid) are metabolized by cyclooxygenase (COX) and LOX into pro-inflammatory eicosanoids (prostaglandins, leukotrienes), amplifying neuroinflammation [174, 175]. Enzymes like DGAT, PCYT1, and ATGL further accelerate aberrant lipid turnover, promoting membrane destabilization. Elevated BMI (>30) exacerbates these pathways through dual mechanisms: Adipose-derived leptin resistance elevates systemic free fatty acids, fueling neuronal lipid peroxidation; and peroxisome proliferator-activated receptor (PPAR)-γ dysfunction in obese microglia amplifies pro-inflammatory eicosanoid synthesis (e.g., PGE_2_). Human lipidomics studies confirm that high BMI correlates with elevated 4-hydroxynonenal (4-HNE), a lipid peroxidation marker, in post-stroke patients [176]. Preclinical models corroborate this, showing 40% larger infarct volumes in high-fat-diet mice with increased astrocytic lipid droplet accumulation [175].

Glutamate excitotoxicity strains this system by activating mGluR receptors, driving PLC-mediated PIP2 hydrolysis into DAG and IP_3_ (Fig. 4b) [174]. This initiates acute lipid reprogramming where phospholipase A2 activation releases free fatty acids and lysophospholipids, triggering rapid membrane destabilization and inflammatory signaling. The resulting reactive oxygen species and cytotoxic aldehydes (e.g., 4-HNE) disrupt ion homeostasis, accelerating subacute ferroptosis through enhanced ALOX15-dependent peroxidation of polyunsaturated fatty acids. Paradoxically, while lipids drive toxicity, they simultaneously enable repair: astrocytes and microglia scavenge lipids to buffer damage, while chronic Lipin-1-mediated membrane repair mechanisms support phospholipid remodeling and structural recovery.

Ischemic stroke thus unveils competing metabolic adaptations where glycolytic overdrive and lipid dysregulation converge, a storm that both devastates and drives recovery. Therapeutic promise lies in phase-specific interventions: targeting acute lipid droplet dynamics, fine-tuning subacute SFA balance to counter ALOX15/GPX4-dependent peroxidation, which propagates membrane damage via Fenton chemistry, or intercepting pro-inflammatory mediators [177, 178]. As single-cell analyses confirm ferroptosis through ACSL4 upregulation and mitochondrial shrinkage [179, 180], the challenge remains to harness lipid metabolism’s regenerative capacity while suppressing peroxidation cascades, thereby advancing targeted neural repair strategies.

## Metabolic reprogramming counteracting neuroinflammation post-stroke

### Lactate as a neuroprotective energy substrate

Lactate, traditionally viewed as a metabolic byproduct, emerges as a critical modulator of neuroinflammation and neuroprotection post-stroke. Experimental studies demonstrate that L-lactate administration (100 mM, 2 µL) into the cerebral ventricles 30 min post-MCAO reduces infarct volume, neuronal apoptosis, and functional deficits [181]. Mechanistically, lactate stabilizes HIF-1α, repressing pro-inflammatory microglial markers such as a cluster of differentiation (CD)-86, inducible nitric oxide synthase (iNOS), IL-6, and NF-α while enhancing anti-inflammatory mediators such as arginase-1, CD206, Chitinase 3-like 3 (Chi3l3, a marker for anti-inflammatory M2 microglia/macrophages), transforming growth factor (TGF)-β, and IL-10 via NF-κB suppression [182] (Fig. 2b). Transcriptomic profiling in oxygen-glucose deprivation-treated BV2 microglia identifies CCL7 as a lactate-responsive chemokine. Recombinant CCL7 administration reverses lactate’s neuroprotective effects, confirming the HIF-1α–CCL7 axis as central to lactate-mediated immunomodulation [136]. Lactate accumulation lowers extracellular pH, thereby stabilizing HIF-1α in microglia. This stabilization suppresses CCL7 expression, which in turn inhibits NF-κB activation, silencing TNF-α and IL-6 production while promoting anti-inflammatory M2 polarization. This process is amplified by systemic lipid mediators: Adipose-derived FFAs cross the compromised BBB, fueling lipid peroxidation and NLRP3 inflammasome activation [50]. Gut-derived TMAO heightens platelet reactivity and cerebral thrombosis, synergizing with lactate to exacerbate neuroinflammation [183]. Microglial pro-inflammatory polarization is further sustained by dysregulated potassium (KCNJ2) and calcium (TRPM2) channels, creating a vicious cycle of cytokine release and immune activation.

### Activation of the pentose phosphate pathway

In ischemic stroke, surviving cells confront a metabolic paradox: generating ATP while defending against oxidative catastrophe and rebuilding macromolecules. The pentose phosphate pathway (PPP) transitions from a routine metabolic route to a critical orchestrator of survival [184]. Post-stroke, proliferating neurons and glia adopt a Warburg-like metabolic shift, prioritizing glycolysis over oxidative phosphorylation to rapidly harvest ATP and funnel intermediates into the PPP, a strategy more commonly associated with cancer cells [185]. This reprogramming is not merely an energy grab but a calculated survival tactic. The PPP’s first arm generates NADPH, the lifeblood of antioxidant defense, which resuscitates glutathione to neutralize the deluge of ROS unleashed by ischemia. Simultaneously, its second arm synthesizes ribose-5-phosphate, the backbone of nucleotides required for DNA repair and the proliferation of repair-oriented cells, from neural progenitors to endothelial cells rebuilding damaged vasculature.

Key enzymes like glucose-6-phosphate dehydrogenase surge in neurons, sustaining redox balance when mitochondrial TCA cycling fails [186]. Glycolysis regenerates NAD^+^ while feeding the PPP and serine/glycine one-carbon metabolism, enabling nucleotide synthesis and epigenetic adaptations. However, this lifeline diverts glucose from ATP production, escalating reliance on anaerobic glycolysis and acidosis risk. Critically, the PPP intersects with systemic inflammation. Amid lipid peroxidation cascades (“lipid storms”), adipose-derived free fatty acids and gut-generated TMAO amplify neuroinflammation and thrombosis [183] (Table S3). The PPP thus becomes a nexus where energy crisis, oxidative stress, and lipid toxicity converge. Targeting PPP flux through NADPH synthesis to quench peroxidation or nucleotide tuning could tip recovery balances, but precision is vital to avoid exacerbating metabolic exhaustion.

Amidst the “lipid storm” of peroxidation and membrane breakdown, the PPP’s role transcends mere intermediary metabolism. It becomes a nexus where energy crisis, oxidative stress, and regenerative demand converge, offering both hope and vulnerability. Targeting PPP flux, enhancing NADPH synthesis to quench lipid peroxidation, or fine-tuning nucleotide production, could tip the balance toward recovery. However, this pathway’s duality as a defender against ROS while also having the potential to contribute to metabolic exhaustion demands precision. As we unravel the PPP’s choreography in the ischemic brain, we confront a fundamental truth: survival following stroke depends not on the function of any single pathway, but on the coordinated and dynamic interplay of metabolic networks that adapt, protect, and facilitate recovery under extreme stress.

### Metabolic reprogramming in peripheral nerve repair

The remarkable capacity of the peripheral nervous system (PNS) to regenerate after injury unveils a masterclass in metabolic adaptation that holds profound implications for understanding recovery in the ischemic brain. At the heart of this process lie Schwann cells, the PNS’s metabolic architects, which coordinate dynamic energy reprogramming to activate regenerative programs essential for axonal regrowth [187]. Central to their strategy is MCT1, a linchpin in lactate shuttling that bridges glycolysis in Schwann cells to mitochondrial respiration in regenerating axons. Disrupting MCT1 stalls this metabolic symbiosis, delaying repair by crippling lipid metabolism and energy production, a stark reminder of lactate’s dual role as both a metabolite and a signaling molecule [170]. Beyond facilitating energy exchange, Schwann cells orchestrate peripheral nerve regeneration through epigenetic reprogramming: inhibition of HDACs amplifies neuregulin signaling via PI3K-AKT/ERK pathways [103, 115], activating transcriptional programs for remyelination. Simultaneously, mTOR-1 fine-tunes protein synthesis to enable dedifferentiation into a pro-regenerative state [188]. This mTOR-driven anabolic shift, while adaptive in peripheral nerves, exhibits divergent outcomes in the central nervous system, where chronic mTORC1 activation in astrocytes drives pathological lipid synthesis and droplet accumulation via DGAT1/PLIN2 upregulation, directly linking mTOR to maladaptive glial reactivity and impaired recovery in chronic stroke phases [35, 189]. Satellite glial cells, often overshadowed by their Schwann counterparts, play a vital role similar to that of astrocytes in the central nervous system by ramping up lipid metabolism to supply axons with fatty acids for membrane rebuilding. This lipid-centric repair strategy echoes the “lipid storm” observed post-stroke, where dysregulated lipid signaling wreaks havoc; however, in the PNS, it is harnessed constructively. Beyond local metabolism, the gut-brain axis casts a surprising influence [190]. Intermittent fasting, through gut microbiota-derived metabolites like indole-3-propionate, recruits neutrophils to dorsal root ganglia, where they clear debris and prime the microenvironment for regeneration. This systemic crosstalk underscores a universal truth: nerve repair is not confined to the injury site but is a whole-body metabolic endeavor.

PNS thus offers a blueprint for metabolic resilience where glycolysis, lipid dynamics, and epigenetic plasticity converge to overcome injury. For ischemic stroke, these lessons are tantalizing. Could enhancing lactate shuttling in astrocytes, modulating HDACs to rewire glial epigenetics, or harnessing gut-derived metabolites to temper neuroinflammation similarly propel recovery in the brain? Regeneration might not be a passive process but a metabolic campaign, demanding precise resource allocation and cross-tissue coordination.

## Therapeutic targeting of metabolic-immune crosstalk

### Mitochondrial modulation

**Fig. 5:**
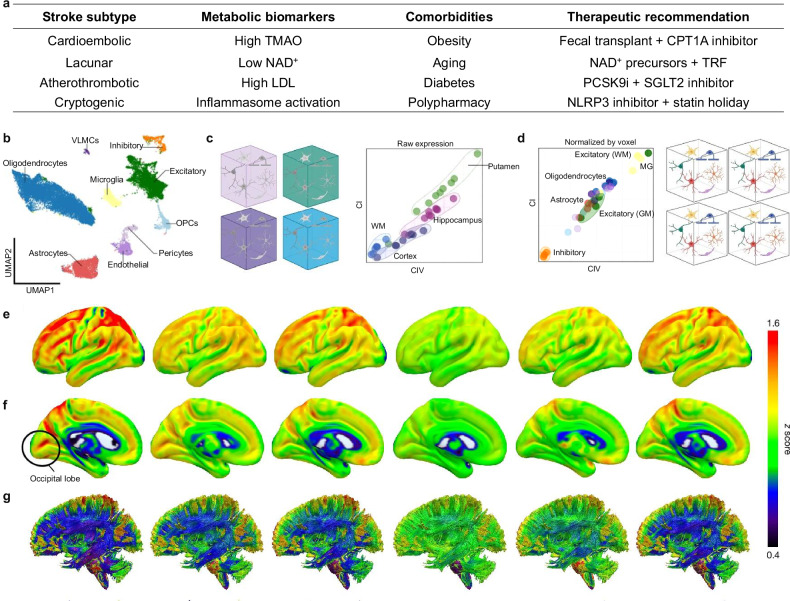
Cell-type-specific metabolism. **a** Stroke subtype-specific metabolic dysregulation and targeted interventions. TMAO: trimethylamine N-oxide; TRF: time-restricted feeding; PCSK9i: proprotein convertase subtilisin/kexin type 9 inhibitor; SGLT2: sodium-glucose cotransporter-2. **b** Uniform manifold approximation and projection plot identifies nine major cell types, including neurons, oligodendrocyte precursor cells (OPCs), and vascular leptomeningeal cells (VLMCs). Cell-type proportions vary by voxel type (GM/WM), with excitatory neurons dominating GM voxels (high OXPHOS demand) and OPCs enriched in WM. **c** Predictive neuroimaging, such as magnetic resonance imaging-based mitochondrial respiratory capacity (MRC) mapping, now stratifies patients by metabolic vulnerability. BMI-driven inflammation: Adipose inflammation hotspots visualized via FDG-PET, revealing heightened neuroinflammation risk in obese patients (BMI > 30). Aging signatures: Quantified MRC decline in the putamen, correlating with NAD^+^ depletion and impaired energy metabolism. High putamen MRC predicts anaerobic glycolysis-driven lactate overproduction and cognitive fatigue, while low white matter MRC signals delayed axonal injury. These integrated overlays enable precision targeting: suppressing glycolytic surge in cognitively impaired elderly or enhancing axonal support in obese patients with mobility risks [49]. **d** After Z-score normalized correction for regional baseline differences, microglia exhibit discordant CI/CIV ratios, suggesting immunometabolic reprogramming. Excitatory neurons (high CI/CIV) are vulnerable to glutamate excitotoxicity. Microglia (discordant CI/CIV) adopt pro-inflammatory M1 states via NF-κB activation. Astrocytes shift to glycolysis, worsening lactate accumulation and BBB leakage. Predictive neuroimaging of mitochondrial phenotypes for the lateral (**e**), the medial (**f**) surfaces, and the WM connections (**g**). 3D brain maps integrate MRC/MitoD data to predict regional metabolic resilience. High MRC in the putamen correlates with lactate overproduction during anaerobic glycolysis, while low MRC in WM links to delayed axonal injury. Magnetic resonance imaging-predicted MRC maps (**e**–**g**) offer translational tools to stratify stroke patients for metabolic therapies. Reprinted from [49]. Springer Nature Limited. Tf transferrin, STEAP3 six-transmembrane epithelial antigen of the prostate 3, DMT1 divalent metal transporter 1, PUFA polyunsaturated fatty acid, GPX4 glutathione peroxidase 4, BSO buthionine sulfoximine.

In multifocal metabolic dysregulation during ischemic stroke, mitochondria exhibit dual roles as both mediators of energy salvage and sources of oxidative stress. Targeting mitochondrial function presents a promising therapeutic approach that integrates metabolic recovery with immune modulation [191, 192] (Table S4). Stroke subtype-specific interventions address distinct metabolic vulnerabilities: cardioembolic strokes with high TMAO benefit from fecal transplant and CPT1A inhibitors, while lacunar strokes with NAD^+^ depletion respond to NAD^+^ precursors and time-restricted feeding (TRF) (Fig. 5a & Table S5).

Cellular heterogeneity underpins these metabolic differences. The uniform manifold approximation and projection plot identifies nine major cell types, with excitatory neurons dominating gray matter voxels (high OXPHOS demand) and oligodendrocyte precursor cells enriched in white matter (Fig. 5b). After Z-score normalization for regional baselines (Fig. 5c), microglia exhibit discordant CI/CIV ratios indicating immunometabolic reprogramming toward pro-inflammatory M1 states via NF-κB activation, while astrocytes shift to glycolysis, exacerbating lactate accumulation and BBB compromise. Predictive neuroimaging now enables precision stratification of these vulnerabilities (Fig. 5e–g). Magnetic resonance imaging-based mitochondrial respiratory capacity (MRC) mapping reveals: BMI-driven inflammation (fluorodeoxyglucose positron emission tomography hotspots in obese patients, BMI > 30), aging signatures (MRC decline in putamen correlating with NAD^+^ depletion), and regional resilience patterns (high putamen MRC predicting lactate overproduction; low white matter MRC signaling axonal injury).

Central to therapeutic development is PGC-1α, a master regulator of mitochondrial biogenesis. Pharmacological PGC-1α agonists enhance mitochondrial genesis across neural cells, bolstering ATP production while reducing ROS through upregulated antioxidant defenses [193]. This dual action rescues energy-deprived neurons and shifts microglia from destructive M1 to reparative M2 phenotypes. Critically, these interventions require metabolic stratification: NAD^+^ precursors restore plasticity in young brains but fail in aged OXPHOS-compromised systems, while CPT1A inhibitors benefit lean subjects but worsen hepatic steatosis in obesity [194, 195].

Equally vital is preserving mitochondrial membrane integrity via mPTP inhibitors like cyclosporine A. By blocking pathological pore opening, these agents prevent the collapse of membrane potential, cytochrome c release, and caspase-driven apoptosis [196]. This sustains penumbral ATP synthesis and stabilizes astrocytic mitochondria, maintaining lactate shuttling to neurons [197]. However, prolonged mPTP blockade risks trapping damaged organelles, demanding precise dosing.

The mitochondrial-immune interplay reveals a broader truth: stroke recovery requires integrated metabolic-inflammation control. While PGC-1α agonists suppress NF-κB via NADPH synthesis [198] (Fig. 5), and mPTP inhibitors reduce mitochondrial DNA-driven TLR9-driven neutrophil infiltration [199]. However, these strategies exist within a delicate balance. Excessive mitochondrial biogenesis may exacerbate oxygen demand in hypoxic regions, and mPTP inhibition could inadvertently shield damaged mitochondria from mitophagy. Therefore, the future lies in combinatorial approaches pairing PGC-1α activation with mitophagy enhancers or coupling mPTP inhibitors with antioxidants to harmonize metabolic recovery with immune homeostasis. Mitochondrial modulation is not merely a technical fix but a reclamation of cellular equilibrium. It challenges us to view the post-stroke brain not as a battlefield of isolated pathways but as an ecosystem where energy, inflammation, and repair are inextricably linked.

### Amino acid metabolism

In the metabolic tumult of ischemic stroke, amino acids emerge as both mediators of survival and architects of collateral damage, their fates intricately tied to the interplay between glycolytic frenzy and lipid peroxidation [100–102, 200] (Box 2). At the epicenter lies arginine, a double-edged precursor to NO. Regulation of the arginine/NO pathway is paramount: while endothelial NO synthase (eNOS)-derived NO sustains cerebral blood flow by vasodilation, iNOS in activated glia overproduces NO, spawning neurotoxic peroxynitrite that exacerbates lipid peroxidation and mitochondrial dysfunction [201]. Therapeutic strategies aim to tip this balance by preserving eNOS activity to maintain vascular tone while silencing iNOS to curb neuroinflammation. Emerging small molecules (e.g., citric acid) selectively inhibit iNOS or bolster arginase activity, diverting arginine toward polyamine synthesis to support axonal sprouting and membrane repair, thus threading the needle between vascular protection and inflammatory mitigation [202].

Equally critical is tackling glutamate, the harbinger of excitotoxic doom. Beyond its role in neuronal hyperactivation, excess extracellular glutamate fuels oxidative stress by depleting glutathione and amplifying lipid peroxidation. Glutamate scavengers like AA147 offer a dual lifeline: by mopping up synaptic glutamate, they blunt excitotoxicity while paradoxically activating the NRF2-KEAP1 antioxidant axis [203]. This triggers a cascade of cytoprotective genes (glutamate-cysteine ligase for glutathione synthesis, heme oxygenase-1 to degrade pro-oxidant heme), effectively dousing the lipid storm’s flames. Notably, NRF2 activation also reprograms macrophage metabolism, shifting them toward anti-inflammatory phenotypes that synergize with arginine pathway modulation to stabilize the neurovascular unit [204].

However, these strategies exist in metabolic crossfire. The same glycolytic surge that depletes NAD^+^, a cofactor critical for sirtuin-mediated eNOS activation, also starves the urea cycle, perturbing arginine homeostasis [205]. Similarly, lactate accumulation from anaerobic glycolysis acidifies the microenvironment, impairing NRF2 nuclear translocation and blunting AA147’s efficacy. Therefore, amino acid metabolism cannot be targeted in isolation; it demands integration with interventions to normalize pH, restore redox balance, and rewire carbon flux. The promise lies in combinatorial approaches pairing arginine analogs with lactate transporter inhibitors or coupling glutamate scavengers with lipid peroxidation blockers to create a metabolic firewall against stroke’s cascading insults.

In this light, amino acids are not mere metabolic substrates but dynamic signaling nodes where survival and demise converge. Their manipulation offers a path to harmonize the brain’s desperate energy grabs with its fragile need for redox and ionic equilibrium, a balance as delicate as it is vital. As we chart this terrain, it is expected to transform the post-stroke milieu from a metabolic battleground (site of competing metabolic adaptations) into a scaffold where amino acids may orchestrate metabolic repair by modulating both glycolytic flux (e.g., serine-driven one-carbon metabolism) and lipid peroxidation (e.g., cysteine-dependent glutathione synthesis), though combinatorial targeting studies are needed [186, 206].

Box 2 Amino acid dysregulation: a hidden driver of post-stroke behavioral decline While ischemic stroke is often framed as a crisis of glycolytic failure and lipid peroxidation, dysregulated amino acid metabolism emerges as a critical yet underappreciated axis of injury and repair. Glutamate–glutamine cycle collapse, marked by astrocytic EAAT1/2 transporter failure and SNAT3 dysfunction, exacerbates oxidative stress, depleting glutathione reserves and heightening risks of post-stroke fatigue and cognitive impairment. Arginine metabolism’s duality, sustaining blood flow via endothelial nitric oxide synthase (eNOS) while fueling microglial neurotoxicity via inducible NOS (iNOS), may explain why some patients develop post-stroke anxiety despite adequate perfusion. Small-molecule arginase enhancers (e.g., norvaline) redirect arginine toward polyamine synthesis, promoting axonal sprouting in preclinical models. Glycine depletion, common in severe stroke, compromises inhibitory neurotransmission, correlating with hyperarousal and sleep disturbances. Intriguingly, branched-chain amino acids (BCAAs) exhibit paradoxical roles: leucine exacerbates astrocytic lipid toxicity (linked to apathy), while isoleucine enhances ketogenesis, a pathway tied to motivation recovery [200]. Emerging trials target these axes, including arginase enhancers to boost axonal sprouting, and glycine analogs to curb ferroptosis, but their success hinges on addressing socioeconomic disparities in dietary protein access, which skew baseline amino acid pools in vulnerable populations [100–102].

### Glycolysis inhibition

In the dysregulated substrate flux and redox imbalance during ischemic stroke (metabolic chaos), the brain’s compensatory shift toward anaerobic glycolysis (a Warburg-like adaptation) initiates a pathogenic cascade: lactate accumulation drives cytotoxic acidosis while concurrently fueling enzymatic lipid peroxidation, demanding targeted strategies to modulate this maladaptive energetic response [133, 207]. HK2 and LDHA, gatekeepers of glycolytic flux, emerge as high-value targets. Inhibiting HK2 curtails the first committed step of glycolysis, reducing glucose-6-phosphate accumulation and its diversion into the PPP, while LDHA blockade prevents pyruvate-to-lactate conversion, averting extracellular acidosis and mitochondrial fragmentation. Preclinically, these inhibitors reduce infarct volumes and improve functional recovery, not merely by lowering lactate but by reprogramming cellular metabolism: forcing neurons to resurrect oxidative phosphorylation in salvageable penumbral regions and redirecting glucose toward the PPP to bolster NADPH-dependent antioxidant defenses [208, 209].

However, glycolysis inhibition is a metabolic tightrope. While curtailing lactate mitigates lipid peroxidation, sparing membranes from 4-HNE assault, it risks starving cells of ATP in regions where mitochondria are irreparably damaged [210]. This paradox underscores the need for spatiotemporal precision: delivering inhibitors post-reperfusion to avoid compromising acute survival mechanisms while promoting later repair. Intriguingly, LDHA inhibition also disrupts the lactate shuttle, silencing lactate’s role as a signaling molecule that sustains pro-inflammatory microglial activation [211]. The result is a dual boon: reduced excitotoxicity and dampened neuroinflammation, though at the cost of impairing astrocyte-to-neuron metabolic support.

The therapeutic horizon lies in selectivity and synergy. Next-generation HK2/LDHA inhibitors with BBB penetrance and cell-specific targeting (preferential astrocyte vs. neuron action) could minimize off-target energy crises. Pairing these agents with mitochondrial protectors, such as cyclosporine A, or lipid peroxidation inhibitors (e.g., ferrostatin-1), may reconcile the competing demands of energy rescue and oxidative defense. In this metabolic recalibration, glycolysis inhibition transcends mere energy modulation; it becomes a strategic strike against the intersecting crises of glycolytic overdrive and lipid storm, offering a lifeline to neurons teetering between salvage and self-destruction.

### Immunometabolic interventions

The brain’s immune and metabolic systems post-stroke engage in a destructive tango, glial cells and lymphocytes fuel inflammation through maladaptive glycolysis, while lipid peroxidation ravages membranes. Breaking this cycle demands interventions that simultaneously recalibrate immune responses and metabolic flux [212]. Astrocyte reprogramming stands at the forefront: forced expression of neurogenic transcription factors like NeuroD1 or Ngn2 converts reactive astrocytes into functional neurons, a feat that not only replenishes lost circuitry but also silences their pro-inflammatory secretome [213]. By transforming A_1_ astrocytes, chronic producers of MMP-9 and ROS into neurons, this strategy dismantles the inflammatory scaffold that perpetuates BBB leakage and mitochondrial stress, indirectly stabilizing energy metabolism in surviving cells (Fig. 2c).

Parallel efforts target T-cell glycometabolism, where the receptor for RAGE emerges as a linchpin. RAGE signaling in Th17 cells drives a glycolytic surge, propelling their pathogenic differentiation and infiltration into the ischemic brain. Inhibiting RAGE redirects glucose away from glycolysis, suppressing IL-17A production and curtailing neutrophil recruitment, a double victory that dampens neuroinflammation while sparing glucose for reparative processes like axonal ATP synthesis [214]. This metabolic-immune shift mirrors the effects of mitochondrial modulators, as reduced Th17 activity diminishes ROS spillover, easing the burden on neuronal antioxidant systems and preserving mitochondrial membrane integrity.

However, these interventions walk a metabolic tightrope. Astrocyte-to-neuron conversion risks depleting the glial workforce essential for lactate shuttling and glutamate clearance, potentially exacerbating energy crises in penumbral regions. Similarly, RAGE inhibition may inadvertently impair beneficial Treg subsets reliant on glycolytic intermediates for anti-inflammatory function [215]. Therefore, precision is paramount: temporal targeting of NeuroD1/Neurogenin2 (Ngn2) post-acute phase, or RAGE blockade coupled with lactate supplementation, could reconcile immune modulation with metabolic support. The promise lies in their synergy; reprogrammed astrocytes may secrete neurotrophic factors that enhance mitochondrial biogenesis in newborn neurons, while subdued Th17 inflammation reduces lipid peroxidation, creating a milieu where glycolysis and oxidative phosphorylation coexist rather than clash. Immunometabolic interventions, therefore, transcend conventional immunosuppression. They represent a paradigm where immune cells and glia are not merely silenced but metabolically reprogrammed to ally with recovery, a vision where the brain’s post-stroke “lipid storm” is quelled not by brute force but by strategic rewiring, transforming inflammatory arsonists into architects of renewal.

### Nanoparticle delivery systems

In the acute phase of ischemic stroke, where unchecked glycolysis fuels lipid peroxidation and mitochondrial dysfunction, nanoparticle-based delivery systems offer a strategy for spatially targeted intervention. These systems deliver immunometabolic modulators such as succinate directly to lesion-specific cell types, enabling localized metabolic reprogramming. Nanocarriers engineered to cross the compromised BBB represent a shift from generalized anti-inflammatory therapies to precision targeting of hyperactive microglia and neurons [216]. Succinate, a TCA cycle intermediate that accumulates during ischemia, becomes therapeutically valuable when encapsulated [217]. Delivered via pH-sensitive nanoparticles, it is selectively released in the acidic penumbra, where it competitively inhibits mitochondrial SDH, thereby blocking reverse electron transport and reducing ROS, a key trigger of NLRP3 inflammasome activation and lipid peroxidation [218]. Similarly, the immunometabolite itaconate plays a dual role in modulating neuroinflammation. Nanoparticles conjugated with microglia-targeting peptides transport itaconate to phagocytic cells, where it exerts anti-inflammatory effects by upregulating NRF2-mediated antioxidant responses and alkylating KEAP1 to stabilize HIF-1α [219]. This paradoxical action simultaneously promotes glycolytic adaptation and suppresses inflammatory signaling, highlighting nanotherapy’s potential to integrate metabolic and immune modulation.

These nanocarriers are often engineered to respond to stroke-specific environmental cues such as local acidosis, oxidative stress, or vascular leakage, to achieve site-specific payload release, thus avoiding systemic side effects like succinate-induced peripheral inflammation or compromised antibacterial defense from systemic itaconate [220]. However, translation to the clinic faces biological constraints. Human microglia express roughly 50% fewer nanoparticle-targeting receptors, such as triggering receptors expressed on Myeloid cells 2 (TREM2), than rodent models, reducing cellular uptake [221]. Moreover, metabolites like itaconate degrade more rapidly in human CSF, with a half-life of 1.2 h compared to 4 h in mice [222].

Despite these challenges, new designs are showing promise. ROS-sensitive nanocarriers can release itaconate specifically in oxidatively stressed tissues [197], while lipid-coated nanoparticles can hijack immune cells to enter ischemic regions [221]. Key areas for optimization include minimizing hepatic clearance and shielding payloads from enzymatic degradation [223]. A dual-delivery strategy is emerging, such as combining succinate with SDH inhibitors or pairing itaconate with lactate transport activators to simultaneously dampen inflammasome activation and restore mitochondrial metabolism [224]. To mitigate translational risk, phase 0 microdosing studies using ^89^Zr-labeled nanoparticles are proposed to assess human biodistribution prior to efficacy trials [225].

Systemic FAO inhibition with agents like etomoxir can lead to lipid redistribution and hepatic steatosis, as observed in 68% of participants in non-stroke clinical trials (NCT02904226) [226, 227]. Preclinical approaches to reduce hepatotoxicity include nanoparticle encapsulation with hepatocyte-avoidant peptides, which reduce liver uptake by up to 90%, or co-administration with PPARα agonists to enhance lipid clearance [228]. Microbiome-based therapies such as fecal microbiota transplantation (FMT) face regulatory and biological hurdles in stroke populations. The FDA mandates an Investigational New Drug (IND) application for FMT in stroke indications, requiring strain-specific GMP production with an estimated cost of $2.3 million, exclusion of immunocompromised individuals (who represent about 25% of the elderly stroke population), and rigorous monitoring for horizontal gene transfer and potential antibiotic resistance [229]. As safer alternatives, Phase I trials (NCT04756561) are evaluating lyophilized Akkermansia capsules [230, 231]. A persistent technical tradeoff in nanoparticle therapy is the balance between systemic clearance and brain penetration. Particles larger than 20 nm evade hepatic filtration but show brain uptake of less than 1%. Conversely, downsizing to <10 nm improves brain entry but raises toxicity risks, including hippocampal apoptosis in rodent models [232].

Comorbidity-specific stratification is crucial in tailoring therapeutic protocols. LDHA inhibitors pose risks in diabetic patients with unstable pH and should be avoided if lactate exceeds 5 mM, with continuous pH monitoring recommended. DGAT1 inhibitors [111], which influence lipid metabolism, are contraindicated in obese patients with FFA levels above 600 μM [233–235], necessitating FFA screening before initiation. For succinate-loaded nanoparticles, renal impairment should be considered: dosing should be adjusted based on glomerular filtration rate (GFR), and administration avoided if the estimated GFR is below 30 mL/min [236].

## Translational challenges and perspectives in MRIS

The translation of MRIS into therapies confronts a pivotal challenge: human behavior and societal context are inseparable from biology. While preclinical models highlight promising targets such as CPT1A inhibitors to quell neuroinflammation and ACMSD modulators to boost NAD^+^, these interventions often falter in clinical trials, not merely due to interspecies metabolic differences but because human stroke recovery is entangled with aging, lifestyle, and systemic inequities [237, 238]. Where rodents pivot to ketones for post-stroke survival, human astrocytes exploit glycogenolysis to sustain energy homeostasis, a divergence underscoring the peril of extrapolating rodent physiology to human disease [239]. Compounding this, preclinical models overwhelmingly use young, healthy animals, sidestepping the age-related comorbidities (hypertension, diabetes, atherosclerosis) that define clinical stroke populations and profoundly reshape metabolic-immune crosstalk [21, 46, 130, 240–246] (Table 4). This mismatch risks therapies succeeding in idealized settings but failing in the metabolically scarred brains of elderly patients.

**Table 4:** Metabolic reprogramming signatures across comorbidities.

Comorbidity	MRIS features	Clinical consequences	Validating evidence
**Diabetes (T2D)**	Paradoxical glycolysis suppression ↑; AGE-RAGE axis → microglial FAO ↑	3× higher lactate/penumbra ratio; 40% less reperfusion benefit	MRI/PET in 215 patients: Hyperglycemia blunts glycolytic flux despite hypoxia (*p* < 0.001) [46, 241]
**Obesity (BMI** > **30)**	Adipose FFA overflow → astrocytic lipid droplets ↑; Leptin resistance → BDNF ↓	Delayed motor recovery (34% less at 90 days); higher depression risk	SERENADE trial: Serum leptin inversely correlates with cognitive recovery (*r* = −0.71, *n* = 300) [21, 240, 245]
**Aging (>70 years)**	NAD^+^ depletion → SIRT1 ↓; Senescent astrocytes ↑ lipofuscin	60% less penumbral salvage; ferroptosis dominance	Brain bank histology: Lipofuscin+ astrocytes in 80% of aged vs. 5% young strokes [243, 244]
**Polypharmacy**	Statins: FFA ↓ but CoQ10 depletion → OXPHOS ↓; Metformin: Complex I inhibition → lactate ↑	Drug-dependent infarct expansion (e.g., metformin + stroke → 25% larger infarcts)	EUREKA cohort: Polypharmacy (≥ 5 drugs) independently predicts poor recovery (odds ratio = 2.3) [242, 246]

Aging and metabolic comorbidities profoundly reshape MRIS trajectories. In elderly patients (>70 years), NAD^+^ depletion, driven by reduced NAMPT expression, impairs sirtuin-1-mediated mitochondrial biogenesis, diminishing energy reserves in peri-infarct regions by 60% compared to younger individuals [243, 244]. Conversely, high BMI exacerbates neuroinflammation through adipokine dysregulation: leptin resistance suppresses hippocampal BDNF, impairing cognitive recovery, while gut dysbiosis depletes Akkermansia, reducing SCFA production by 40% and compromising BBB integrity [247]. These factors stratify MRIS into distinct phenotypes: elderly diabetics exhibit “pseudo-hypoxic” glycolysis (HIF-1α ↑ /PDK ↑ ), while young obese patients show FAO/NLRP3 dominance, explaining why NAD^+^ boosters benefit aged brains but fail in young diabetics [248].

Emerging solutions address this heterogeneity. Chronobiology reframes therapeutic timing: administering C-C chemokine receptor 2 (CCR2) antagonists aligned with circadian rhythms of immune cell trafficking could enhance efficacy while respecting individual sleep-wake cycles, critical for patients whose work schedules disrupt circadian alignment [249]. Predictive neuroimaging, such as magnetic resonance imaging-based MRC/MitoD mapping, now stratifies patients by metabolic vulnerability: High MRC in the putamen predicts lactate-driven cognitive fatigue, as validated in 45 stroke patients [49]. High MRC in the putamen correlates with anaerobic glycolysis-driven lactate overproduction, while low MRC in WM predicts delayed axonal injury. These 3D brain maps could guide behaviorally tailored therapies targeting anaerobic glycolysis in cognitively impaired subgroups or prioritizing axonal support in those at risk of mobility loss (Fig. 5). Meanwhile, humanized 3D organ-on-a-chip models and brain organoids are dismantling species barriers, recapitulating the neurovascular unit with human cells to decode MRIS in a clinically relevant milieu [250, 251]. These systems reveal how diabetic hyperglycemia exacerbates astrocytic lipid peroxidation or how aged endothelial cells falter in lactate shuttling, insights invisible in conventional models. Complementing this, machine learning algorithms are disentangling the Gordian knot of multi-omics data, integrating genomics, metabolomics, and microbiome signatures to forge personalized MRIS profiles. Imagine clinics where β-hydroxybutyrate levels guide ketogenic diet titration or Akkermansia muciniphila-enriched fecal transplants are prescribed to patients with stroke-induced dysbiosis [183], a future where metabolic therapy is as precise as it is potent.

The path forward demands a reckoning with biology’s complexity. Dynamic metabolic-immune networks, mapped through single-cell metabolomics and spatial transcriptomics, will reveal how gut-derived metabolites sway microglial lipid metabolism or how senescent astrocytes hijack the PPP to resist apoptosis. To mechanistically dissect gut-brain crosstalk in this framework, we propose: (i) gnotobiotic mouse models comparing stroke outcomes in microbiota-depleted versus Akkermansia-reconstituted cohorts to establish causality; (ii) spatial metabolomics mapping SCFA gradients against microglial polarization states in peri-infarct regions; (iii) neutrophil depletion studies testing whether SCFA-mediated neuroprotection requires neutrophil recruitment [252]. Stage-specific interventions should emerge with acute blockade of ferroptotic lipid peroxidation followed by chronic enhancement of mitochondrial biogenesis, all while respecting circadian metabolic rhythms.

### CPT1A to reduce fatty acid oxidation-driven neuroinflammation for neurobehavioral recovery

CPT1A, the rate-limiting enzyme in mitochondrial FAO, is a pivotal therapeutic target in ischemic stroke due to its role in amplifying neuroinflammation. During ischemia, neurons and glia undergo metabolic reprogramming toward FAO to compensate for glycolytic collapse, generating ROS and pro-inflammatory lipid mediators (e.g., arachidonic acid derivatives) that exacerbate neurovascular injury [253]. While CPT1A inhibitors (etomoxir, perhexiline) show preclinical efficacy in reducing infarct volumes and improving motor recovery in rodents by attenuating NLRP3 inflammasome activation and IL-1β release [212], translation to humans faces significant hurdles. Crucially, human hepatocytes exhibit 10-fold higher CPT1A sensitivity than rodent models, explaining dose-limiting hepatotoxicity (ALT elevation in 60% of non-stroke trials) [129]. This species difference necessitates innovative delivery approaches like nanoparticle encapsulation to avoid hepatic exposure and patient stratification using FAO biomarkers. Plasma C16-carnitine >2 μM emerges as a promising stratification biomarker, correlating strongly with neuroinflammation severity [49]. Early clinical trials should further address comorbidity-driven metabolic rewiring, particularly in diabetes, where altered lipid metabolism amplifies neuroinflammatory cascades [254].

### ACMSD modulators to boost NAD^+^ via the kynurenine pathway for cognitive recovery

ACMSD, a pivotal regulator of the kynurenine pathway, offers a novel therapeutic strategy to combat NAD^+^ depletion, a core metabolic defect in stroke driving mitochondrial dysfunction and cognitive decline. Pharmacological inhibition of ACMSD amplifies NAD^+^ synthesis, restoring redox balance and enhancing sirtuin-1-mediated synaptic plasticity essential for post-stroke memory consolidation and emotional regulation [110]. While ACMSD knockdown improves cognitive recovery in aged mice [255], therapeutic translation requires careful calibration: excessive pathway activation risks accumulating neurotoxic quinolinic acid, potentially exacerbating anxiety symptoms [256–263] (Table S6). Critically, no ACMSD-specific drugs have entered clinical trials to date. Current NAD^+^ precursors (e.g., nicotinamide riboside) show limited clinical efficacy in stroke recovery (Phase II: National Institutes of Health Stroke Scale, NIHSS of −1.2 at 90 days) [106], primarily due to poor central nervous system (CNS) bioavailability. To overcome this barrier, next-generation delivery strategies like exosome-encapsulated NAD^+^ or intranasal administration are being explored to enhance brain penetration. These advances raise important ethical considerations: as biomarker-guided metabolic therapies emerge, we should ensure equitable access for elderly populations to prevent widening recovery disparities.

### Time-restricted feeding to align MRIS with circadian metabolic peaks

TRF, which aligns caloric intake with circadian-active phases, exemplifies how metabolic reprogramming intersects with human behavior to shape stroke recovery. By synchronizing peripheral and central circadian rhythms, TRF restores the body’s natural metabolic oscillations, optimizing glucose utilization during active hours and ketogenesis during rest, a process critical for mitigating post-stroke neuroinflammation and mitochondrial dysfunction [264]. In rodent models, TRF not only reduces infarct size but also enhances motor recovery, suggesting that metabolic timing influences neural repair mechanisms [265]. Human pilot trials reveal that TRF improves insulin sensitivity and dampens systemic inflammation [266], yet its success hinges on behavioral adherence, as societal norms around irregular eating schedules and shift work often clash with circadian-aligned feeding. Personalized TRF regimens, adapted to individual chronotypes (e.g., “night owls” vs. “early birds”), could harmonize metabolic benefits with lifestyle preferences, thereby enhancing rehabilitation participation and reducing post-stroke fatigue. However, socioeconomic disparities in meal timing flexibility, such as limited control over work hours or access to nutritious food, risk excluding vulnerable populations from TRF’s advantages. Integrating TRF into stroke care thus demands more than biological insight; it requires addressing the cultural, economic, and psychological barriers that dictate daily eating patterns. When viewed through the MRIS lens, TRF becomes a behavioral-metabolic intervention, where the simple act of when we eat transforms cellular survival strategies into tangible improvements in cognitive function, emotional resilience, and community reintegration.

### Fecal transplants enriched in Akkermansia muciniphila to enhance SCFA production

The gut-brain axis emerges as a critical mediator of post-stroke neurobehavioral recovery, with Akkermansia muciniphila, a keystone gut bacterium, serving as both a metabolic architect and a behavioral modulator [267]. Post-stroke dysbiosis depletes Akkermansia, exacerbating BBB leakage and microglial activation. Fecal microbiota transplantation from Akkermansia-rich donors in mice restores butyrate levels, upregulates cerebral claudin-5 expression, and polarizes microglia toward an anti-inflammatory M2 phenotype [268]. By enhancing SCFA production, Akkermansia fortifies gut barrier integrity, reducing endotoxin-driven neuroinflammation and BBB leakage, processes that directly influence cognitive dysfunction and emotional dysregulation in stroke survivors [269]. Clinical trials reveal that Akkermansia supplementation elevates circulating butyrate levels, correlating not only with improved neurological scores but also with enhanced patient motivation and engagement in rehabilitation, likely via butyrate’s role in dopaminergic signaling [269]. However, this therapeutic promise is tempered by societal and practical challenges: socioeconomic disparities in dietary fiber access limit endogenous Akkermansia abundance in marginalized populations. Synbiotic strategies pairing Akkermansia with culturally adaptable prebiotics (inulin-rich local staples) may democratize access, ensuring microbiome interventions align with diverse dietary practices. Therefore, fecal microbiota transplantation represents a metabolic-behavioral intervention, where restoring gut ecology can recalibrate the brain’s inflammatory tone, turning the gut into an ally in the battle against post-stroke apathy and social withdrawal.

## Conclusion

Mounting evidence compels a paradigm shift: ischemic stroke is not merely a vascular event but a systemic metabolic cascade where the brain’s glycolytic-lipid collision triggers dysregulated crosstalk between peripheral organs and microbial symbionts [1–5, 13, 183]. First, the neurovascular unit serves as a metabolic nexus, modulated by hepatic ketogenesis flooding the bloodstream with neuroprotective β-hydroxybutyrate [10, 51], adipose lipolysis fueling inflammatory storms [12, 130], and skeletal muscle myokines influencing penumbral resilience [188]. Second, circadian biology becomes essential in every therapeutic decision. The rhythmic control of pyruvate dehydrogenase activity by brain and muscle ARNT-like 1 (BMAL1) indicates that a CCR2 antagonist administered at dawn may reduce neuroinflammation, while the same drug given at dusk could worsen it. Third, the gut microbiome functions as both ally and saboteur: its metabolites permeate the BBB to suppress microglial NLRP3 inflammasomes or prime neutrophil infiltration [13, 183, 269]. This complexity demands therapies targeting multi-organ networks, silencing adipose lipolysis while amplifying hepatic ketogenesis, or reseeding gut microbiota to convert loss of metabolic homeostasis (metabolic chaos) into resilience.

This expanded perspective clarifies why decades of neurocentric therapies have not been successful. The same mitochondrial PDK4 isoform that drives neuronal glycolytic overload in the penumbra also regulates cardiomyocyte metabolism during post-stroke heart failure, illustrating that the systemic effects of MRIS are significant. Furthermore, single-cell metabolomics reveals that astrocytes in diabetic stroke models exhibit pathological lipid droplet accumulation, diverting lipid-derived substrates away from neuronal salvage pathways, a metabolic behavior absent in non-diabetic, age-matched controls. These findings emphasize the need for metabolome-resolved clinical trials, where CRISPR-edited organoids model patient-specific mutations in ACMSD to enhance NAD^+^ salvage, while spatial lipidomics can track the dissemination of arachidonic acid cascades from peri-infarct neurons to distant renal tubules.

### Precision through metabolic phenotyping

MRIS is dynamically remodeled by comorbidities, aging, and genetic variation, necessitating biomarker-driven stratification [85–96]. Comorbidity-specific signatures include: diabetic stroke with paradoxical glycolysis suppression and accelerated lipid peroxidation [185]; hypertensive stroke featuring microvascular FAO exhaustion and impaired nitric oxide signaling [122, 130]; aging-related blunted ketone utilization (Stroke Preclinical Assessment Network, SPAN trial: 60% lower β-hydroxybutyrate uptake) [47, 48, 255]; and obesity (BMI > 35) suppressing PPP activity and glutathione reserves [141–143]. Genetic variants critically modulate therapeutic responses: APOE ε4/ε4 impairs lipid efflux, favoring DGAT1 inhibitors over CPT1A inhibitors [270]; solute carrier family 2, member A (SLC2A1 rs710218) reduces glucose transporter type 1 (GLUT1) activity, contraindicating LDHA inhibitors [271]; NAMPT rs2302559 compromises NAD^+^ salvage, necessitating higher-dose NAD^+^ precursors; and CPT1A rs2229738 enhances FAO flux, prohibiting CPT1A inhibitors [272] (Table 5).

**Table 5:** Genetic variants influencing MRIS therapy response.

Gene	Variant	Metabolic impact	Therapeutic implication
*APOE*	ε4/ε4	Impaired lipid efflux → lipid droplets	DGAT1 inhibitors >CPT1A inhibitors
*SLC2A1*	rs710218	Reduced GLUT1 activity → glycolysis ↓	Avoid LDHA inhibitors
*NAMPT*	rs2302559	Impaired NAD^+^ salvage	Higher-dose NAD^+^ precursors
*CPT1A*	rs2229738	Enhanced FAO flux	Contraindicated if plasma C16-carnitine >2 μM

Multidimensional metabolic phenotyping represents a transformative approach, integrating spatial lipidomics to map arachidonic acid cascades [49], CSF panels quantifying lactate, itaconate, and NAD^+^ ratios [106, 273], and machine learning algorithms that synthesize plasma biomarkers with neuroimaging and genomic data [49]. This enables precision interventions: DGAT1 inhibitors for APOE ε4 carriers with astrocytic lipid droplets [90, 98, 99, 270], or NAD^+^ salvage for aged SIRT1-deficient penumbras with NAMPT variants [244, 245]. Concurrently, safety-aware designs should address hepatic monitoring for FAO inhibitors (etomoxir elevates liver triglycerides by 40%) [274]; cancer risk stratification for NAD^+^ boosters in SLC2A1 variant carriers [275]; and immunosuppression screening for fecal transplants [276].

### Chrono-optimized and convergence-ready therapeutics

A comprehensive approach to MRIS therapy integrates biomarker-driven patient stratification, circadian-informed treatment timing, and the engineering of peripheral-central metabolic crosstalk to enhance stroke recovery. Precise identification of metabolic subphenotypes enables the selection of interventions that match individual pathophysiology, while chronotherapeutic strategies align treatment delivery with endogenous metabolic rhythms to maximize efficacy and minimize side effects (Table S7). By combining these approaches with modulation of peripheral organ systems, this framework can transform stroke management from isolated vascular treatment to a coordinated, systemic metabolic rehabilitation.

#### Biomarker-driven stratification

The heterogeneity of MRIS metabolic subphenotypes necessitates biomarker-driven stratification to overcome the limitations inherent in homogeneous stroke therapies. Priorities include spatial lipidomics mapping of 4-HNE gradients to identify ferroptosis-vulnerable penumbral regions, CSF panels quantifying lactate, itaconate, and NAD^+^ ratios [106, 73], and machine learning integration of plasma β-hydroxybutyrate levels with mitochondrial respiratory capacity mapping [49]. Clinical translation, however, faces four key challenges. First, temporal variability in biomarkers such as lactate peaking at 6 h post-stroke and normalizing by 24 h, alongside 40% diurnal fluctuations in NAD^+^, can be addressed through circadian-adjusted sampling and the use of subdermal microsensors [24, 277]. Second, sample accessibility is limited, with CSF collection contraindicated in approximately 30% of anticoagulated patients; this barrier may be overcome by leveraging exosome-enriched plasma mitochondrial DNA (mtDNA) with a 70% increase in sensitivity or salivary metabolomics, where lactate and glucose levels correlate strongly with CSF measurements (*r* = 0.81) [49]. Third, analytical costs remain prohibitive, but these expenses may be mitigated by the use of low-cost paper-based lactate and β-hydroxybutyrate tests, complemented by AI-enhanced MRI techniques predicting lipid peroxide accumulation with high accuracy (AUC = 0.91) [49]. Fourth, validation gaps persist, with 89% of proposed biomarkers lacking multicenter rigor; these deficiencies are being addressed by the RAPID-BIOS consortium through standardized protocols and comorbidity-adjusted reference ranges. Implementing tiered biomarker panels beginning with rapid first-tier plasma lactate and β-hydroxybutyrate assays for acute triage, followed by second-tier CSF and metabolomic profiling for non-responders, enables precision therapy matching. This approach facilitates personalized interventions such as DGAT1 inhibitors for obese patients exhibiting astrocytic lipid droplet accumulation [278], or NAD^+^ boosters for elderly patients with impaired ketone utilization, as exemplified in the SPAN trial [47, 48, 255].

#### Chronotherapeutic optimization

The circadian clock plays a central role in orchestrating metabolic reprogramming during ischemic stroke through the transcriptional regulation of key metabolic pathways. Core clock genes modulate MRIS dynamics by coordinating energy metabolism across the day: BMAL1/CLOCK complexes activate genes such as CPT1A, enhancing hepatic FAO at dawn, and NAMPT, driving peak NAD^+^ synthesis at dusk. REV-ERBα suppresses PDK4 expression at night to reduce glycolytic lactate accumulation, while PER2 regulates astrocytic lipid droplet formation through DGAT1 transcriptional control [278]. These molecular rhythms define circadian therapeutic windows that have been validated in preclinical models. Administration of the CPT1A inhibitor etomoxir during the active phase (ZT14–22) reduced infarct volume by 42% in mice, double the efficacy observed during the rest phase, by aligning with PPARα/BMAL1-driven optimization of FAO [279]. Similarly, nicotinamide riboside given at rest onset (ZT12) doubled cognitive recovery in rats by synchronizing with peak SIRT1 activity and mitochondrial biogenesis rhythms [280], while time-restricted feeding (7:00–15:00) improved motor recovery by 37% in aged mice through coordinated hepatic ketogenesis and β-oxidation [281].

Emerging human studies support these circadian dependencies. In the CIRCLE-STROKE trial (NCT04845165), CCR2 antagonists administered between 6:00 and 8:00 reduced IL-6 and TNF-α levels by 51% more than twice the effect of evening dosing. Similarly, metabolic syndrome patients adhering to an 8:00–16:00 feeding window exhibited a 57% increase in ketogenesis and insulin sensitivity, both critical for post-stroke metabolic resilience [282]. These findings underscore four core principles of chronotherapeutic MRIS management: Dawn BMAL1 activation upregulates hepatic CPT1A, driving peak FAO at sunrise, a rhythm exploited by administering CPT1A inhibitors at dawn to maximally suppress pathological lipid flux; dusk NAMPT expression peaks optimize NAD^+^ precursor efficacy; nighttime PER2-mediated NOX4 suppression enables antioxidant repair; and feeding cycle-synced SCFA production reduces morning endotoxemia [42, 190, 264]. Accordingly, MRIS therapies should be precisely timed to align with endogenous metabolic rhythms, administering FAO inhibitors at dawn when BMAL1 activity is highest or NAD^+^ boosters at dusk to coincide with NAMPT expression peaks. These strategies can be further personalized using wearable ketone monitors for shift workers and tailored feeding windows based on stroke subtype, enabling a time-sensitive reprogramming of post-ischemic metabolic recovery.

#### Peripheral-central crosstalk engineering

Peripheral organs play a critical role in shaping the course of ischemic stroke and offer therapeutic leverage in MRIS-targeted interventions. Advances such as humanized multi-organ chips now model how diabetic hyperglycemia exacerbates astrocytic lipid droplet accumulation, while Akkermansia-targeted fecal microbiota transplants promote SCFA-mediated BBB stabilization [283]. In parallel, hepatocyte-directed PPARα gene therapy has been shown to enhance systemic β-hydroxybutyrate availability, providing a neuroprotective energy substrate during cerebral metabolic crisis [128, 284, 285].

MRIS therapies enhance rather than replace vascular interventions like thrombectomy by stabilizing the metabolic environment. FAO inhibitors support endothelial integrity post-recanalization, while NAD^+^ boosters preserve mitochondrial function during sustained hypoperfusion [6–8, 129]. Implementation requires comorbidity-adjusted precision: patients with BMI > 35 exhibit suppressed pentose phosphate pathway activity and glutathione depletion, rendering them vulnerable to free fatty acid overload and unsuitable for CPT1A inhibition [141–143]. Critically, polypharmacy interactions demand coordinated management (Table S8): Statins deplete coenzyme Q10, impairing OXPHOS and increasing ferroptosis risk, necessitating CoQ10 supplementation (~100 mg/day) [286]; metformin inhibits complex I, driving compensatory glycolysis and lactate acidosis, warranting withholding for 48 h post-stroke with lactate monitoring [287]; sodium-glucose cotransporter-2 (SGLT2) inhibitors elevate ketogenesis, which may provide neuroprotective β-hydroxybutyrate but risks ketoacidosis, requiring euglycemia maintenance and β-hydroxybutyrate (β-OHB) monitoring [288]; and anticoagulants deplete vitamin K, impairing Gas6-mediated myelin repair, thus indicating vitamin K2 supplementation. Moreover, polypharmacy itself poses independent risks. Drug combinations like metformin during stroke can expand infarct size by 25%, and the European research on kinetics in acute stroke cohort shows polypharmacy (≥5 drugs) predicts poor recovery (odds ratio = 2.3) [242, 246]. Translational risks further require proactive management, including hepatic monitoring for FAO inhibitors, cancer screening with NAD^+^ augmentation, and rigorous donor screening for fecal microbiota transplantation.

Neglecting this complexity risks repeating the failures of one-size-fits-all stroke therapies. An international MRIS consortium integrating metabolomics, chronobiology, and digital health is urgently needed to develop biomarker-validated, chrono-optimized interventions grounded in systems-level pathophysiology [289]. The challenge of MRIS lies not in suppressing metabolic activity but in restoring its equilibrium, moderating the lipid storm’s destructive cascade while amplifying ketogenesis-driven resilience. By redefining stroke as a systemic metabolic blueprint, we might design therapies that recalibrate maladaptive survival mechanisms into coordinated programs of recovery. Stroke treatment is evolving toward harmonized brain-peripheral metabolic crosstalk, where targeted silencing of adipose lipolysis, amplification of hepatic ketogenesis, and strategic reseeding of the gut microbiome collectively convert metabolic dysregulation into resilience.

## Supplementary information

Supplementary file for publication
