# Supplementary material for: Metabolic reprogramming in ischemic stroke: when glycolytic overdrive meets lipid storm
Source: Cell Death Dis. 2025 Nov 3;16(1):788. doi: 10.1038/s41419-025-08114-w (PMC12583499; doi:10.1038/s41419-025-08114-w)
Supplement: Supplementary file 1 — Supplementary file for publication [file 41419_2025_8114_MOESM1_ESM.pdf]

Supplementary Information

Metabolic reprogramming in ischemic stroke: when glycolytic overdrive meets lipid storm

Yuchun Wang[1,2,3], Minyan Ge[1], Jinling Wang[1], Yiming Xu[1,2,3], Nianhong Wang[1,2,3,], Shumao Xu[1,]**

[1]Department of Rehabilitation Medicine, Huashan Hospital, Institute of Science and Technology for Brain-inspired Intelligence (ISTBI), Fudan University, Shanghai, 200040, China

[2]National Center for Neurological Disorders, Shanghai 200040, China

[3]National Clinical Research Center for Geriatric Diseases, Shanghai 200040, China

[*]Correspondence to: wnh@fudan.edu.cn; shumaoxu@fudan.edu.cn

Supplementary Table 1. Impact of age and BMI on MRIS biomarkers

Biomarker	Aging effect	Obesity effect	Clinical implication
**Lactate**	↑ Accumulation (impaired MCT1)	↑ Glycolytic flux	False-positive penumbra detection
**β-Hydroxybutyrate**	↓ Hepatic synthesis	↓ Ketogenesis (insulin resistance)	Reduced neuroprotection
**Succinate**	↑ Microglial production	↑ Adipocyte release	Exaggerated neuroinflammation

Supplementary Table 2. The role of amino acids in physiology and post-stroke alterations

Amino acid	Essential?	Functions in stroke	Pathways/receptors	Post-stroke alterations	Clinic applications
**Excitotoxicity**					
Glutamate	No	Drives neuronal hyperexcitability via NMDA/AMPA receptors; Ca^{2+} overload → mitochondrial dysfunction.	NMDA receptor, mGluR, mitochondrial permeability pore	↑ Extracellular accumulation (excitotoxicity)	NMDA antagonists (memantine), EAAT2 enhancers
Aspartate	No	Modulates NMDA receptor activity; precursor for N-acetylaspartate (NAA), critical for neuronal lipid synthesis.	NMDA receptor, NAA synthase	↓ Aspartate and NAA in infarct core	NAA supplementation in preclinical models
**Neuroinflammation**					
Tryptophan	Yes	Kynurenine pathway dominates: → Quinolinic acid (NMDA agonist) vs. kynurenic acid (NMDA antagonist).	IDO1, KMO, KYNU	↑ Kynurenine:Quinolinic acid ratio in severe stroke	IDO1 inhibitors (e.g., epacadostat)
Phenylalanine	Yes	↑ Phenylalanine → competitive inhibition of tyrosine hydroxylase → reduced dopamine	Tyrosine hydroxylase, phenylalanine hydroxylase	↑ Serum levels correlate with infarct volume	Dietary restriction in acute phase

Amino acid	Essential?	Functions in stroke	Pathways/receptors	Post-stroke alterations	Clinic applications
		synthesis.			
**Oxidative stress**					
Cysteine	No	Rate-limiting substrate for glutathione (GSH) synthesis; ↓ GSH → lipid peroxidation and ferroptosis.	Glutamate-cysteine ligase (GCL), System Xc^{-}	↑ Serum levels (compensatory) but ↓ GSH in neurons	NAC (N-acetylcysteine) to boost GSH
Methionine	Yes	↑ Methionine → hyperhomocysteinemia → endothelial dysfunction and NLRP3 inflammasome activation.	Methionine synthase, CBS	↑ Serum methionine and homocysteine	Folate/B12 supplementation to lower homocysteine
**Neuroprotection**					
Glycine	No	Co-agonist of NMDA receptors; paradoxically neuroprotective at extrasynaptic sites. Precursor for glutathione.	GlyR, NMDA receptor	↓ Acute-phase urine glycine; CSF glycine correlates with recovery	Glycine receptor agonists (D-cycloserine)
Serine	No	Supports one-carbon metabolism (via SHMT) for nucleotide synthesis; precursor for D-	Serine hydroxymethyltransferase (SHMT), DAO	↓ Serum levels in ischemia; SHMT reversed in diabetes	L-Serine supplementation in preclinical models

Amino acid	Essential?	Functions in stroke	Pathways/receptors	Post-stroke alterations	Clinic applications
		serine (NMDA co-agonist).			
**Energy metabolism**					
Leucine	Yes	Activates mTORC1 → inhibits autophagy; exacerbates lipid droplet accumulation in astrocytes.	mTORC1, Sestrin2	Variable (↑ in plasma but ↓ cerebral levels)	mTORC1 inhibitors (rapamycin)
Isoleucine	Yes	Ketogenic AA; fuels β-oxidation in astrocytes during glucose deprivation.	PPARα, CPT1A	↑ Plasma levels in fasting → neuroprotection	Ketogenic diet adjunct
Valine	Yes	Modulates BCAA transaminase → glutamate/glutamine cycling.	BCAT1/2	↓ Cerebral levels in chronic ischemia	BCAA-restricted diets in metabolic syndrome
**Vascular & immune**					
Arginine	No	Substrate for eNOS (vasodilation) vs. iNOS (peroxynitrite). Arginase competes → polyamines (axonal repair).	eNOS, iNOS, arginase	↑ Serum arginine but ↓ bioavailability due to ADMA	Citrulline supplementation to bypass hepatic arginase
Histidine	Yes	Precursor for	Histidine	↑ Cerebral	H1

Amino acid	Essential?	Functions in stroke	Pathways/receptors	Post-stroke alterations	Clinic applications
	(infants)	histamine → regulates microglial activation and BBB permeability.	decarboxylase, H1/H4 receptors	histamine post-stroke → vasogenic edema	antagonists (cetirizine) in preclinical studies

Supplementary Table 3. Vascular processes modulated by metabolism

Vascular mechanism	Metabolic influence	Clinical impact
**Thrombosis**	Platelet PFKFB3-driven glycolysis fuels clot contraction	Diabetic hyperglycemia → 40% larger thrombi
**Reperfusion injury**	Succinate accumulation during ischemia → ROS burst at reperfusion	Serum succinate >5 µM predicts hemorrhagic transformation (OR=3.1)
**Neurovascular coupling**	Astrocytic lactate shuttling regulates arteriolar tone	Lactate >4mM disrupts functional hyperemia (fMRI)
**BBB integrity**	Endothelial FAO ↑ reduces occludin glycosylation	FAO inhibitors restore BBB in hyperlipidemic models

Supplementary Table 4. Therapeutic agents targeting metabolic-immune crosstalk

Target	Mechanism	Agents	Adverse effects	Mitigation strategies	Comorbidity considerations
**CPT1A**	Inhibits fatty acid oxidation	Etomoxir	Hepatic steatosis (↑ liver TG 40%); Cardiac hypertrophy (impaired ATP synthesis)	Liver-targeted siRNA; BMI-dependent dosing (avoid if BMI>35)	Avoid in MASLD/obesity (hepatic risk)
**Lactate dehydrogenase**	Suppresses glycolytic flux	GSK2837808A, FX11	Systemic acidosis	Alkalinization protocols	Caution in diabetes (hyperglycemia risk)
**HIF-1α**	Stabilizes hypoxia response	Roxadustat, FG-4592	Off-target angiogenesis (VEGF↑)	MRI tumor screening	Contraindicated in cancer history
**PARP-1**	Preserves NAD^+ for mitochondrial repair	Olaparib, Veliparib	DNA repair interference	Transient dosing windows	Avoid in DNA repair disorders
**DGAT1**	Blocks lipid droplet formation	Pradigastat	Severe diarrhea (30% patients)	Enteric-coated formulations	Monitor in IBD patients
**NLRP3 inflammasome**	Suppresses IL-1β release	MCC950, OLT1177	Broad immunosuppression	Short-course pulsed therapy	Avoid in active infections
**mPTP**	Maintains mitochondrial membrane integrity	Cyclosporine A	Nephrotoxicity (elderly)	Renal function monitoring	High risk in CKD/hypertension
**Mitochondrial ROS**	Scavenges superoxide radicals	MitoQ	Ineffective in hyperglycemia	Pair with SGLT2 inhibitors	Limited efficacy in diabetes
**NAD^+ salvage**	Boosts sirtuins	Nicotinamide riboside	Flushing, tumorigenesis risk	Extended-release	Prioritize for elderly (>65 years)

Target	Mechanism	Agents	Adverse effects	Mitigation strategies	Comorbidity considerations
	activity			formulations	
**Gut microbiome**	Restores SCFA production	Akkermansia-FMT	Bacteremia (4.3%), engraftment failure	Synthetic consortia (GMP-grade)	Contraindicated in immunosuppressio n

Supplementary Table 5. Stroke subtype-specific metabolism

Subtype	Metabolic vulnerability	Targeted therapy
**LAA**	LDL-driven endothelial FAO ↑	PCSK9 inhibitors + DGAT1
**Cardioembolic**	TMAO → platelet hyperactivity	TMA lyase inhibitors + anticoagulants
**Lacunar**	BBB leakage → SCFA deficiency	Butyrate nanoparticles
**Hemorrhagic transformation**	MMP-9 ↑ → lipid peroxidation ↑	Ferroptosis inhibitors (liproxstatin-1)

Supplementary Table 6. Clinical translation assessment

Therapeutic strategy	Preclinical evidence	Clinical trials	Challenges	Biomarker bridge
**CPT1A inhibition**	40% infarct reduction in rodents (etomoxir)[1]	Phase I (NCT04562805: etomoxir safety)	Hepatic toxicity; diabetic comorbidity interference[2]	Plasma acylcarnitines (FAO flux) + MRI lipid peroxidation mapping
**ACMSD modulation**	Cognitive recovery in aged mice (ACMSD-KD)[3]	None (NAD^+ precursors in Phase II: NR NCT04809966)	Neurotoxic quinolinic acid risk; blood-brain barrier penetration[4]	CSF quinolinic acid/kynurenine ratio + fMRI executive function
**Nanoparticle metabolites**	55% infarct reduction (succinate NPs in rats)[5]	None (no BBB-penetrant nanocarriers approved)	Immune clearance; metabolite stability[6]	PET imaging of NP biodistribution + CSF itaconate levels
**Fecal microbiota transplant**	30% neuroscore improvement (Akkermansia-FMT in mice)[7]	Phase I stroke trials (NCT04756561, NCT04127591)	Standardization; long-term engraftment[8]	Serum butyrate + gut metagenomics + BBB permeability (dynamic CT)

Supplementary Table 7. Targeted metabolic therapies with chronotherapeutic optimization

Target	Intervention	Mechanism	Preclinical outcome	Clinical status	Regulators	Circadian optimization
Glycolysis	LDHA inhibitor (GSK2837808A)	Blocks lactate production → reduces acidosis	↓ Infarct size (35%), ↑ motor recovery (Mice)	Phase I (NCT04873622)	AMPK↑, HIF-1α↓	Not yet established
FAO	CPT1A inhibitor (Etomoxir)	Inhibits fatty acid oxidation → ↓ lipid toxicity	↓ Neuroinflammation, ↑ cognitive function (Rats)	Phase II (NCT04562805)	AMPK↓, mTOR↓	Dawn (6:00-8:00): 42% ↓ infarct vs. evening
NAD^+ Salvage	Nicotinamide riboside	Boosts NAD^+ → activates SIRT1/PGC-1α	↑ Mitochondrial biogenesis, ↓ neuronal death	Phase III (NCT05143996)	SIRT1↑, PARP-1↓	Dusk (18:00-20:00): 2× ↑ cognitive recovery
mTOR	Rapamycin	Inhibits mTORC1 → ↓ lipid synthesis	Attenuates astrocytic lipid droplets	Preclinical	mTOR↓, ULK1↑	Not yet established
Feeding Schedule	TRF (16:8 fasting)	Synchronizes hepatic ketogenesis/β-oxidation	↑ Motor recovery (37%) in aged mice	Phase II (NCT04933968)	BMAL1↑, PER2↓	8:00-16:00 eating: 37% ↑ motor recovery

Supplementary Table 8. Polypharmacy interactions

Drug	Metabolic interaction	MRIS impact	Clinical management
**Statins**	HMGCR inhibition → CoQ10 ↓	Impaired OXPHOS; ↑ ferroptosis risk	CoQ10 supplementation (100 mg/day)
**Metformin**	Complex I inhibition	Compensatory glycolysis ↑ → lactate acidosis	Withhold 48h post-stroke; monitor lactate
**SGLT2 inhibitors**	Ketogenesis ↑	Neuroprotective BHB ↑; but ketoacidosis risk	Maintain euglycemia; monitor β-OHB
**Anticoagulants**	Vitamin K depletion ↓	Reduced γ-carboxylation of Gas6 → impaired myelin repair	Vitamin K2 supplementation

Abbreviations

Abbreviation	Full Name	Context/Notes
**ACMSD**	Aminocarboxymuconate Semialdehyde Decarboxylase	Enzyme in tryptophan/kynurenine pathway
**ADMA**	Asymmetric Dimethylarginine	eNOS inhibitor
**AMPA**	α-Amino-3-hydroxy-5-methyl-4-isoxazolepropionic acid	Glutamate receptor type
**BBB**	Blood-Brain Barrier	
**BCAA**	Branched-Chain Amino Acids	Leucine, Isoleucine, Valine
**BCAT1/2**	Branched-Chain Aminotransferase 1/2	Enzymes
**BHB**	β-Hydroxybutyrate	Ketone body
**BMI**	Body Mass Index	
**BMAL1**	Brain and Muscle ARNT-Like 1	Core circadian clock protein
**CBS**	Cystathionine Beta-Synthase	Enzyme in homocysteine metabolism
**CoQ10**	Coenzyme Q10 (Ubiquinone)	Mitochondrial electron carrier
**CPT1A**	Carnitine Palmitoyltransferase 1A	Enzyme for mitochondrial fatty acid import
**CSF**	Cerebrospinal Fluid	
**CT**	Computed Tomography	Imaging
**DAO**	D-Amino Acid Oxidase	Enzyme
**DGAT1**	Diacylglycerol O-Acyltransferase 1	Enzyme for triglyceride synthesis
**EAAT2**	Excitatory Amino Acid Transporter 2	Major glutamate transporter
**eNOS**	Endothelial Nitric Oxide Synthase	Enzyme producing NO (vasodilator)
**FAO**	Fatty Acid Oxidation	

Abbreviation	Full Name	Context/Notes
**FMT**	Fecal Microbiota Transplant	
**fMRI**	functional Magnetic Resonance Imaging	
**GCL**	Glutamate-Cysteine Ligase	Rate-limiting enzyme for glutathione synthesis
**GMP**	Good Manufacturing Practice	Quality standard
**GSH**	Glutathione	Major antioxidant
**HIF-1α**	Hypoxia-Inducible Factor 1-alpha	Transcription factor activated by low oxygen
**HMGCR**	3-Hydroxy-3-Methylglutaryl-CoA Reductase	Rate-limiting enzyme in cholesterol synthesis (target of statins)
**IBD**	Inflammatory Bowel Disease	Comorbidity
**IDO1**	Indoleamine 2,3-Dioxygenase 1	First enzyme in kynurenine pathway (tryptophan catabolism)
**iNOS**	Inducible Nitric Oxide Synthase	Enzyme producing NO (pro-inflammatory)
**KMO**	Kynurenine 3-Monooxygenase	Enzyme in kynurenine pathway
**KYNU**	Kynureninase	Enzyme in kynurenine pathway
**LAA**	Large Artery Atherosclerosis	Stroke subtype
**LDHA**	Lactate Dehydrogenase A	Isoform converting pyruvate to lactate
**LDL**	Low-Density Lipoprotein	"Bad" cholesterol
**MASLD**	Metabolic Dysfunction-Associated Steatotic Liver Disease	Formerly NAFLD
**MCT1**	Monocarboxylate Transporter 1	Lactate transporter
**mGluR**	Metabotropic Glutamate Receptor	
**MMP-9**	Matrix Metalloproteinase-9	Enzyme degrading extracellular matrix (contributes to

Abbreviation	Full Name	Context/Notes
		BBB breakdown)
**mPTP**	Mitochondrial Permeability Transition Pore	Channel whose opening triggers cell death
**mTOR**	Mechanistic Target of Rapamycin	Protein kinase regulating growth, metabolism
**mTORC1**	mTOR Complex 1	
**NAA**	N-Acetylaspartate	Marker of neuronal health/viability
**NAD^+**	Nicotinamide Adenine Dinucleotide	Coenzyme central to energy metabolism & signaling
**NAC**	N-Acetylcysteine	Precursor for glutathione synthesis
**NCT**	National Clinical Trial (identifier prefix)	
**NLRP3**	NLR Family Pyrin Domain Containing 3	Inflammasome sensor component
**NMDA**	N-Methyl-D-Aspartate	Glutamate receptor type
**NP**	Nanoparticle	
**OR**	Odds Ratio	Statistical measure (e.g., OR=3.1)
**OXPHOS**	Oxidative Phosphorylation	Mitochondrial ATP production
**PARP-1**	Poly(ADP-Ribose) Polymerase 1	DNA repair enzyme; consumes NAD^+
**PER2**	Period Circadian Regulator 2	Core circadian clock protein
**PCSK9**	Proprotein Convertase Subtilisin/Kexin Type 9	Protein regulating LDL receptor degradation (target of inhibitors)
**PET**	Positron Emission Tomography	Imaging
**PFKFB3**	6-Phosphofructo-2-Kinase/Fructose-2,6-Biphosphatase 3	Enzyme regulating glycolysis rate
**PPARα**	Peroxisome Proliferator-Activated Receptor Alpha	Transcription factor regulating fatty acid oxidation/ketogenesis

Abbreviation	Full Name	Context/Notes
**ROS**	Reactive Oxygen Species	
**SCFA**	Short-Chain Fatty Acids	Microbial metabolites (e.g., butyrate)
**SHMT**	Serine Hydroxymethyltransferase	Enzyme in one-carbon metabolism
**siRNA**	Small Interfering RNA	Gene silencing tool
**SGLT2**	Sodium-Glucose Cotransporter 2	Renal glucose reabsorption transporter (target of inhibitors)
**TMAO**	Trimethylamine N-Oxide	Gut microbiota-derived metabolite linked to thrombosis
**TG**	Triglycerides	
**TRF**	Time-Restricted Feeding	Eating within a specific daily window
**ULK1**	Unc-51 Like Autophagy Activating Kinase 1	Kinase initiating autophagy
**VEGF**	Vascular Endothelial Growth Factor	Protein stimulating blood vessel growth

Reference

1. Maarman, G., Marais, E., Lochner, A. & Du Toit, E.F. Effect of chronic CPT-1 inhibition on myocardial ischemia-reperfusion injury (I/R) in a model of diet-induced obesity. *Card. Drugs Therapy* **26**, 205-216 (2012).
2. Xie, Y. et al. CPT1A protects podocytes from Lipotoxicity and apoptosis in Vitro and Alleviates Diabetic Nephropathy in vivo. *Diabetes* **73**, 879-895 (2024).
3. Palzer, L. et al. Alpha-amino-beta-carboxy-muconate-semialdehyde decarboxylase controls dietary niacin requirements for NAD+ synthesis. *Cell Rep.* **25**, 1359-1370. e1354 (2018).
4. Brundin, L. et al. An enzyme in the kynurenine pathway that governs vulnerability to suicidal behavior by regulating excitotoxicity and neuroinflammation. *Translat. Psych.* **6**, e865-e865 (2016).
5. Li, J., Yuan, Z., Liu, H., Feng, J. & Chen, Z. Size-dependent tissue-specific biological effects of core–shell structured Fe_3O_4@ SiO_2–NH_2 nanoparticles. *J. Nanobiotech.* **17**, 1-14 (2019).
6. Wang, B., He, X., Zhang, Z., Zhao, Y. & Feng, W. Metabolism of nanomaterials in vivo: blood circulation and organ clearance. *Acc. Chem. Res.* **46**, 761-769 (2013).
7. Zhang, P.-p. et al. Fecal microbiota transplantation improves metabolism and gut microbiome composition in db/db mice. *Acta Pharmacol. Sin.* **41**, 678-685 (2020).
8. Aggarwala, V. et al. Precise quantification of bacterial strains after fecal microbiota transplantation delineates long-term engraftment and explains outcomes. *Nat. Microbiol.* **6**, 1309-1318 (2021).
